# A survey of UK and Irish veterinary and veterinary nursing Students’ engagement with small animal nutrition information

**DOI:** 10.1371/journal.pone.0351963

**Published:** 2026-07-16

**Authors:** Rachel H. Lumbis, Samantha J. Fontaine, John J. Reilly, Euan D. Bennet, Philippa S. Yam

**Affiliations:** 1 College of Medical, Veterinary and Life Sciences, School of Biodiversity, One Health and Veterinary Medicine, University of Glasgow, Glasgow, United Kingdom; 2 Department of Psychological Sciences and Health, Physical Activity and Health Group, University of Strathclyde, Glasgow, United Kingdom; Kerman University of Medical Sciences, IRAN, ISLAMIC REPUBLIC OF

## Abstract

The global prevalence of diet-related non-communicable disease is rising, along with popularity of unconventional and alternative diets and the availability of unsubstantiated information. Considerable confusion and misinformation exist regarding nutrition and dietary choices. The veterinary healthcare team needs to be a trusted and primary source of sound nutritional advice and be able to signpost clients to appropriate nutrition information. This study’s objective was to establish the sources of nutrition information used by first-year veterinary students (VS) and veterinary nursing students (VNS) in the UK and Republic of Ireland. Participants were recruited by non-probability, convenience purposive sampling, by email invitation from educational providers. Data were collected between October 2023 and January 2024. Participation was voluntary and informed consent obtained. 135 VS and 186 VNS completed the online survey. Most (82%, n = 211) expressed an interest in learning about small animal nutrition. Qualified veterinarians were perceived to be more knowledgeable about pet nutrition (73%, n = 270) than veterinary nurses (53%, n = 132). Veterinary healthcare professional and journal articles ranked most valued sources of information, with media, social media, and friends/family least valuable. Students considered evidence-based information for dietary decisions by pet caregivers (93%, n = 213) to be important, yet only 53% (n = 138) felt sufficiently knowledgeable to access evidence-based nutritional information. This investigation highlights the paradox that trusted nutrition information sources are not always the most used. This insight into the nutrition information-seeking behaviour of student veterinary professionals can help inform educators in the provision of skills and knowledge to evaluate information and utilise trustworthy sources.

## Introduction

The humanisation of animals and current-day notion of pets as family members is a key trend [1] which, together with numerous developments in companion animal nutrition, is driving the pet food market [2]. A wide array of pet food is now available, with growth in the availability of conventional and unconventional diets. The global growth in the pet food market continues, mirroring the increase in pet population, with nutrition-related support considered fundamental to disease prevention and management [3–9]. Food is cited as the greatest pet-related expenditure [10]. Pet caregivers are considered to be informed consumers [11] with preferences for how, and from whom, information is delivered. The use of marketing strategies, health claims and unregulated terms, combined with caregivers’ inability to accurately interpret pet food labels [12,13] can make appropriate pet food selection challenging, with many finding this the most difficult aspect of pet ownership [14]. Furthermore, the amount of nutrition-related information is extensive and varies in terms of quality and accuracy. Several factors, including accessibility, trustworthiness and the human-animal bond, are considered to influence and affect pet caregivers’ choice of information source and information search behaviour [15–19]. Yet, few pet caregivers have the ability to assess the reliability, accuracy and credibility of information [15,20] or to understand and interpret this [21,22]. Resultant low nutrition literacy, with potential confusion, misinformation and conflicting advice, can result in the feeding of suboptimal, or even dangerous diets, with deleterious effects on pet health and wellbeing [17,23–25] and can undermine trust in veterinary professionals and evidence-based practice [22,26].

Correct nutrition is a key factor in optimising animal health, wellbeing, performance, quality of life and longevity; a reflection of its recognition as the fifth vital assessment of a standard physical examination for small animals [27,28]. Nutrition intersects with virtually every aspect of veterinary medicine [29] but is often an overlooked and underdiscussed aspect of veterinary care [30–33]. Many pet caregivers desire nutrition-related advice from a veterinary professional [34–36], but it is considered a highly emotive and controversial subject [37,38]. Other veterinary-related factors include limited time [30,35,39,40], lack of veterinarian knowledge or confidence [30,31], staff inconsistency and lack of practice policy [41]. Client-related factors include misinformation [40,42] information overload [35], and caregivers’ concerns over cost and the financial motivation of veterinarians [38,43]. These combined issues mean discussions surrounding nutrition are infrequent or avoided.

Several studies have explored pet caregivers’ use of, and trust in, information sources [14,16,22,35,44–46], however few have identified the preferred sources of information used and trusted by student veterinarians and veterinary nurses to learn more about small animal nutrition [47,48]. It is a key responsibility of the veterinary healthcare team to be a trusted and primary source of sound nutritional advice [14] and to be able to signpost clients to appropriate sources of nutrition information [15,49]. Student veterinary professionals enter their studies with preconceived notions about small animal nutrition (50). Preferred information sources can potentially inform and misinform, ultimately impacting professional practice, patient care, client education and the ability to foster strong relationships with pet caregivers [50,51]. It is therefore essential that the training of student veterinary professionals is sufficient to provide the skills and knowledge to educate pet caregivers about diet choice and to critically evaluate information and promote the utilisation of appropriate information sources. Completion of a nutritional assessment is considered a day one competency for newly qualified veterinarians and registered veterinary nurses in the UK and Ireland [52–55]. Yet, whilst awareness of the concept is apparent, this is often neglected and seldom completed in full at every veterinary visit. Reasons for this disconnect are numerous and include under-representation of nutrition-related education in veterinary curricula [56], veterinary professionals’ self-perceived deficiency in skills, competence and knowledge and understanding of fundamental nutrition principles [57–60] and confidence to discuss nutrition with pet caregivers [61], especially when trying to recommend dietary change [30,62].

This manuscript presents results of a study to evaluate the small animal nutrition-related competence of first year veterinary students (VS) and veterinary nurse students (VNS). This study was conducted as part of a broader two-part project to compare the competence of foundation and professional phase students. Study findings that focus on students’ perceptions towards nutrition care and education have been presented and discussed in a previous publication [63]. The primary aim of the current study was to establish the sources of nutrition information used and trusted by first year (foundation phase) student veterinary professionals. Secondary objectives were to ascertain if foundation phase VS and VNS discuss pet nutrition with qualified veterinary professionals and to identify how knowledgeable they perceive them to be about it.

## Methods

A cross-sectional study of UK and Irish VS and VNS was conducted. Ethical approval was granted by the University of Glasgow, College of Medical, Veterinary & Life Sciences Ethics Committee (reference number 200230001).

### Study participants

At the time of the study, there were 12 veterinary schools, 11 in the UK and one in Ireland, 14 awarding universities with full or provisional Royal College of Veterinary Surgeons (RCVS) accreditation and five universities with Veterinary Council of Ireland (VCI) accreditation offering, respectively, UK and Ireland-based higher education qualifications in veterinary nursing. Contact was made with all institutions, as well as with the two veterinary nursing further education training providers in Ireland and all three UK-based veterinary nursing further education awarding organisations. According to data published by the Veterinary Schools Council and UCD School of Veterinary Medicine websites, there were 1,850 first year veterinary students in the UK and Ireland. According to the RCVS, there were 1,360 student veterinary nurse enrolments between September and December 2023. The reported annual cohort size for each VCI-accredited veterinary nursing programme was obtained from publicly available sources and used as a proxy for the number of training places available nationally (240). Using Cochrane’s formula, the number of responses (135 VS and 186 VNS) from this maximum potential population resulted in a post-hoc margin of error (95% confidence level) of 6.5% for VS, and 5.4% for VNS respectively. Eligibility criteria required participants to be VS or VNS, aged 18 years or over and entering their first year of study in 2023, in the UK or Republic of Ireland. Participants were recruited by non-probability, convenience purposive sampling. Consistent information was emailed to all academic institutions, together with a PowerPoint slide containing an anonymous questionnaire link via QR code and an explanation of the research. No incentive was offered to research participants.

### Questionnaire design

An online survey enabled the collection of a large sample size, offered better management of respondent selection and facilitated follow up comparisons to be made (Evans and Mathur, 2018). A series of short answer, multiple-choice, and free response questions and structured question statements capturing perceptions on a 5-point Likert scale, were included as part of a broader 62-item anonymous online questionnaire (Supporting Information files) to ascertain VS and VNS’ self-reported proficiency, knowledge, and attitudes towards small animal nutrition [63]. For the purposes of this study, small animal was defined as companion animals or pets, kept for domestic purposes rather than for food production. This novel questionnaire was developed by the lead author (RL) and was informed by similar research conducted in the veterinary [64] and human healthcare [65–70] disciplines. It comprised sections on: (a) demographics (age, gender, college/university, postcode); (b) pet ownership; (c) nutrition-related information and experience; (d) students’ perceptions of the relevance and importance of nutrition-related education; and (e) students’ self-perceived relevance of, and confidence in, nutrition-related patient care and pet caregiver advice. The focus of the current study was to identify the sources of nutrition information used and trusted by first year student veterinary professionals. Students were also asked to rate the small animal nutrition-related knowledge of qualified veterinary professionals and self-perceived attitudes towards nutrition-related education. As such, a relevant subset of responses are presented in the current manuscript and, specifically, in relation to questions 1–3 (section a), 5, 6, 8–11 (section b), 13–17 (section c), 24, 26, 27 (section d), 33–35 (section e, VNS questionnaire) and 36–38 (section e, VS questionnaire). Despite non-identical numbering, questions within section e were identical for each student group. Questions were answered if relevant to the participant and, where applicable, responses reflected the species of pet owned or cared for.

Methodological advice was provided by the institution’s Lecturer in Research and Numerical Skills (EB). Content validity was confirmed through two stages of pre-testing. Ten volunteers of varying age, gender, pet caregiver status and veterinary experience piloted the survey in the initial stage, ensuring the final version was well-constructed, unambiguous, and capable of eliciting accurate and meaningful data. An online pre-test of the questionnaire was then conducted with another small group of volunteers. All applicable suggestions that were likely to improve data quality were considered and incorporated into the final version. Estimated time for completion was up to 15 minutes. The reliability of the questionnaire was assessed using Cronbach’s alpha.

### Data collection

Following pre-testing, the questionnaire was distributed to VS and VNS via Qualtrics® (Provo, Utah) by email invitation from each education provider. Use of an anonymous link and adjusted settings to prevent the collection of respondents’ IP address and location data ensured the provision of anonymous responses. The survey was set up to prevent multiple responses from the same individual. Survey data was collected between 2^nd^ October 2023 and 1^st^ January 2024. Participation was voluntary and informed consent was obtained by clicking to ‘agree’ with consent statements prior to entering the questionnaire.

### Data analysis

Responses were downloaded into Microsoft® Excel® for Microsoft 365 MSO (Version 2405) and, where applicable, assigned numerical values for easier computation. Statistical analysis was performed using the IBM SPSS software, version 29.0.1.0 (171). Where applicable, data was analysed with descriptive statistics. Agreement with statements were indicated by a response of 4 or 5 on a 5-point Likert scale (1 = strongly disagree, 5 = strongly agree); disagreement was indicated by a response of 2 or less. Categorical data were reported as counts and proportions and analysed using Pearson’s Chi-square test, or Fisher’s exact test when greater than 20% of cells had expected frequencies under five. Statistical significance was set at P < 0.05.

Free text responses were analysed thematically [71] utilising a small q methodology within a framework of (post)positivism [72,73]. The analysis was inductive, identifying themes occurring within the data as opposed to researchers’ knowledge of the subject area or underpinning theory.

## Results

### Demographics

Responses were received from 135 veterinary students (VS) and 186 veterinary nursing students (VNS). A respective 82% (n = 111) of VS and 89% (n = 166) of VNS identified as female, with 15% (n = 20) of VS and 5% (n = 10) of VNS identifying as male. Most (61%, n = 194) of the respondents were ≤20 years old, with 35% (n = 113) aged between 21–30, 4% (n = 13) aged between 31–40 and less than 1% (n = 1) aged over 40. Seven out of twelve veterinary schools were represented, with the most VS respondents from the Universities of Glasgow, Edinburgh and Bristol. VNS respondents represented 17 veterinary nurse training providers, with eight offering a higher education programme, eight offering a further education route and one offering both.

### Pet ownership

**Fig 1:**
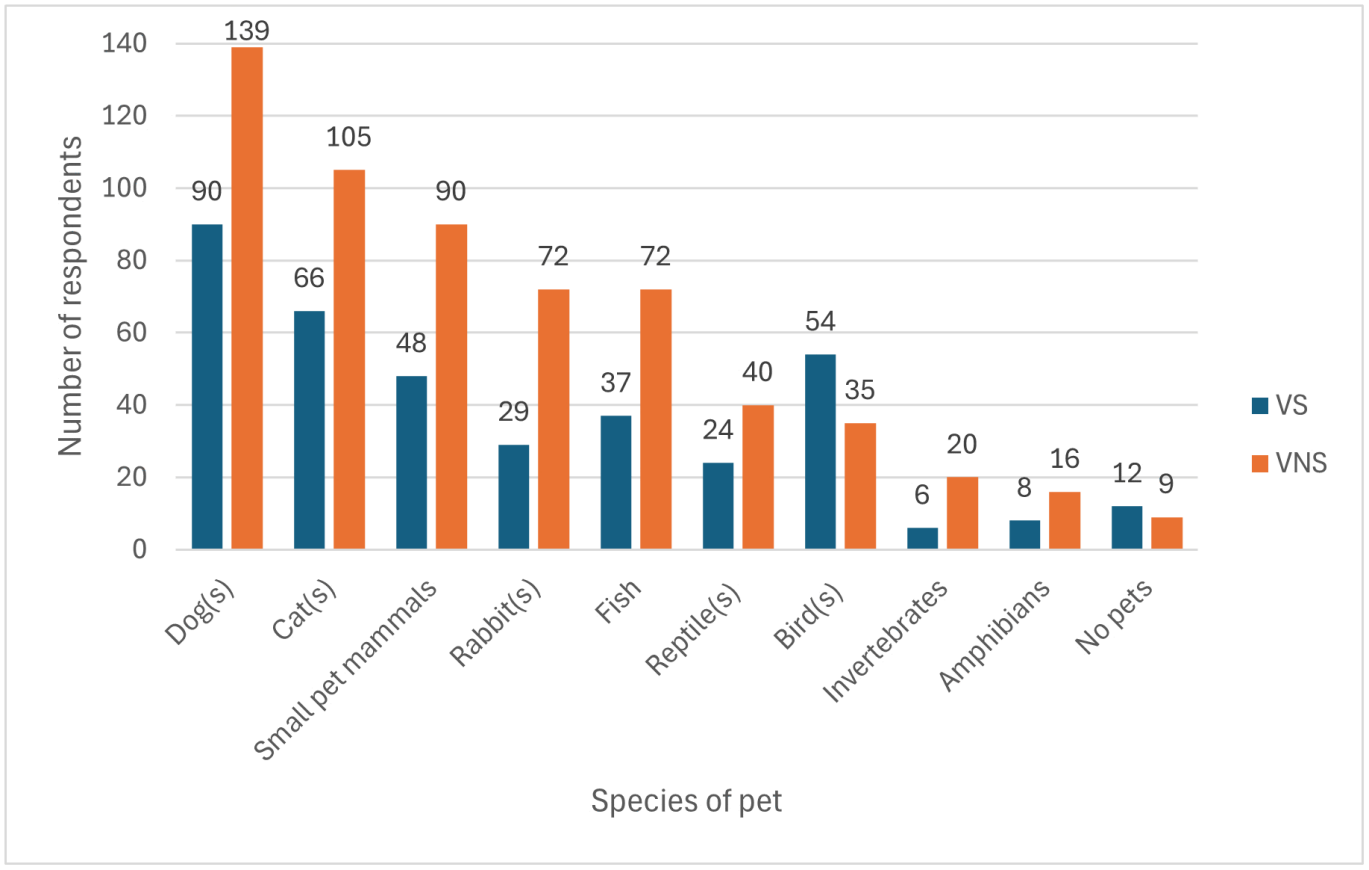
Number of respondents with responsibility for the food selection and management of small pet species.

**Fig 2:**
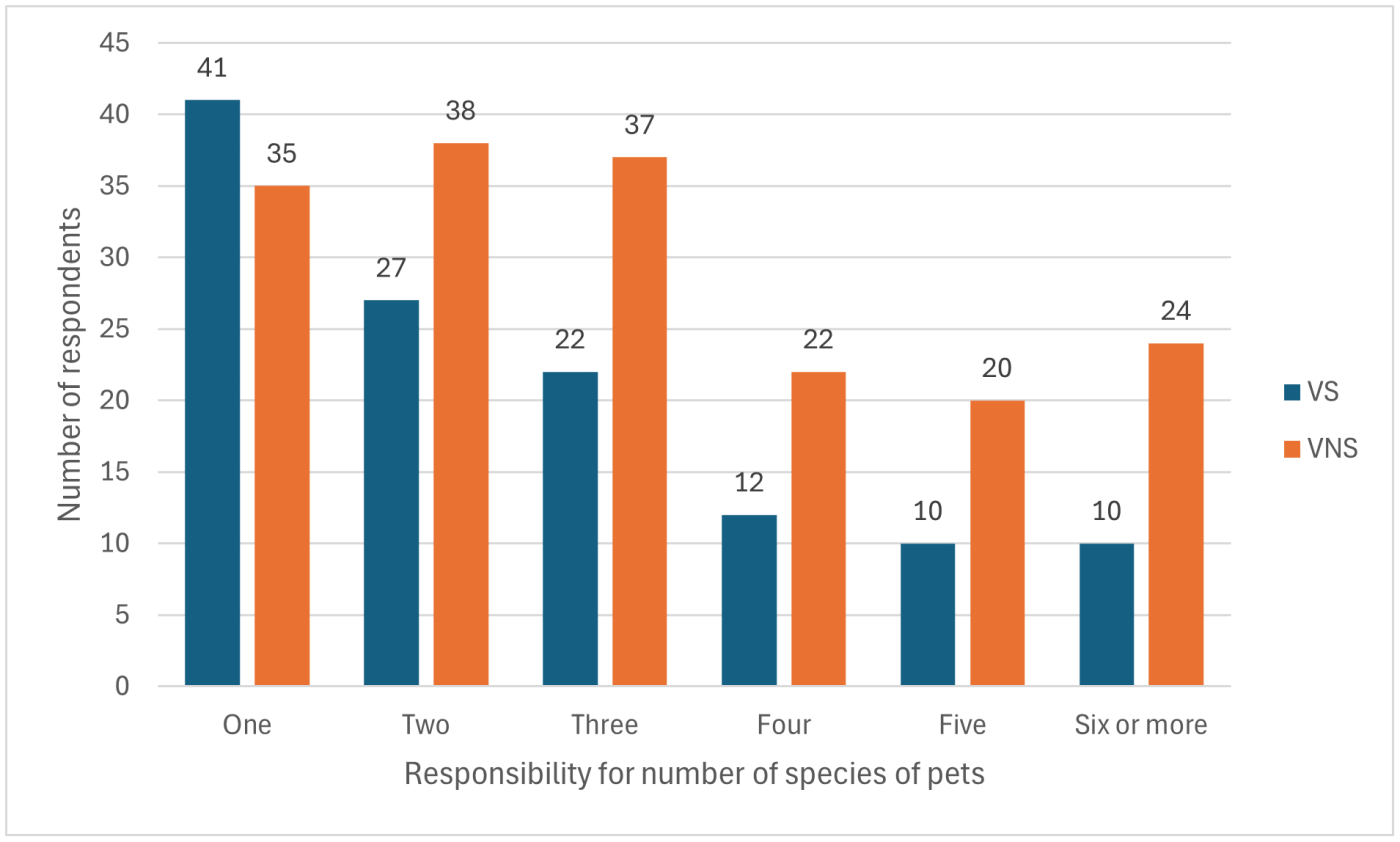
Number of respondents with responsibility for the food selection and management of one or more species or type of small animal pet.

**Fig 3:**
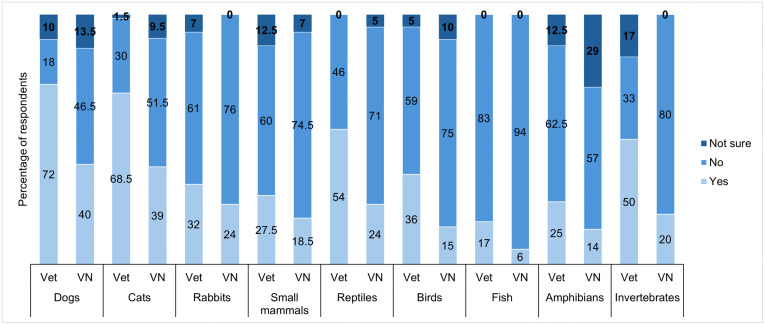
The percentage of veterinary students who have discussed their pet’s nutrition with a qualified veterinarian (vet) and veterinary nurse (VN).

**Fig 4:**
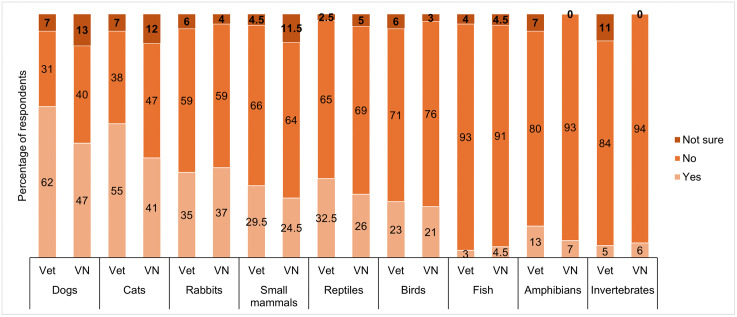
The percentage of veterinary nursing students who have discussed their pet’s nutrition with a qualified veterinarian (vet) and veterinary nurse (VN).

**Fig 5:**
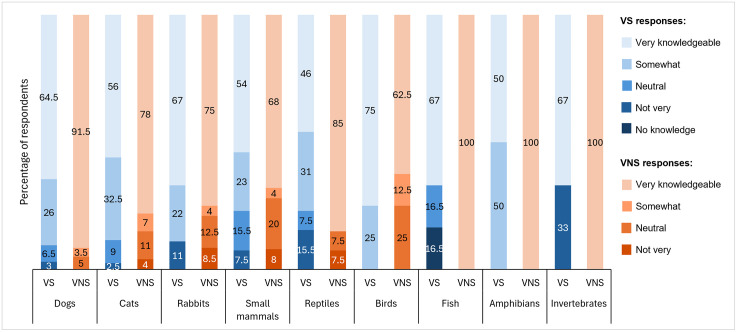
Nutrition knowledge of qualified veterinarians as perceived by veterinary students (VS) and veterinary nurse students (VNS).

**Fig 6:**
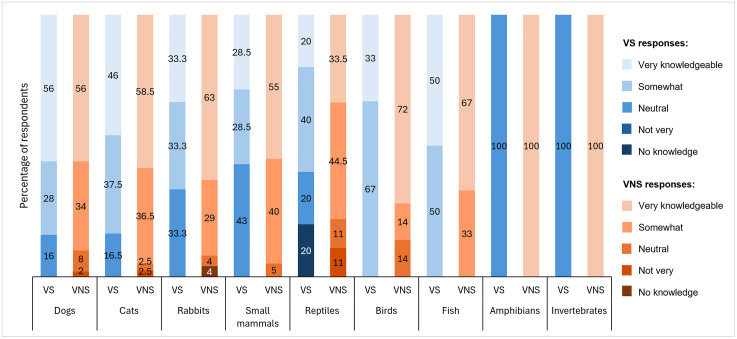
Nutrition knowledge of qualified veterinary nurses as perceived by veterinary students (VS) and veterinary nurse students (VNS).

Nearly all students had responsibility for the food selection and management of one or more pets (Fig 1), but a greater number of VNS than VS had responsibility for multiple pet species (Fig 2). This difference was not statistically significant (Chi-squared test p = 0.1). VNS and VS were more likely to consider their dogs, cats, rabbits, small mammals, reptiles and birds to be family members than their fish, amphibians and invertebrates. Overall, a higher percentage of student veterinary professionals had discussed the nutrition of their dogs, cats, small mammals, reptiles and birds with a qualified veterinarian than a veterinary nurse (Figs 3 and 4). A respective 72% and 69% of VS and 62% and 55% of VNS discussed the nutrition of their dog(s) and cat(s) with a veterinarian. A reduced number (40% and 39% of VS and 47% and 41% of VNS) discussed the nutrition of their dog(s) and cat(s) with a veterinary nurse. More VNS than VS had discussed their pets’ nutrition with a qualified veterinary nurse, but this difference was not statistically significant (Chi-squared test p = 0.7). Both student groups were statistically less likely to have discussed the nutrition of their fish (p < 0.015) and invertebrate (p < 0.021) with a vet. Veterinarians were perceived to have a greater level of knowledge about pet nutrition (73%, n = 270) than veterinary nurses (53%, n = 132) (Figs 5 and 6) but the nutrition knowledge of veterinary nurses was rated higher by VNS than VS. Most student veterinary professionals perceived veterinarians to be very knowledgeable about canine (p < 0.001) and feline (p < 0.010) nutrition.

### Perceived relevance and importance of nutrition-related education

Most students (82%, n = 211) expressed interest in learning about small animal nutrition. The majority of VS (73%, n = 73) and VNS (88%, n = 139) had confidence in the ability of their nutrition-related education to adequately prepare them for work as a veterinary professional. Fisher’s Exact Test determined a significant association (two-tailed p = 0.005) between student type and confidence that their nutrition training will prepare them for work as a veterinary professional, with both VNS and VS strongly skewed towards agree and strongly agree. This viewpoint was supported by several open text responses:

“Vets have an extended knowledge of nutrition.” (VS58)

“People in the veterinary profession would be most knowledgeable about an animal’s nutrition and weight” (VS63)

“Vets and tech’s are qualified professionals in their field. Part of their qualification will include a deep knowledge of nutrition, and their accreditation allows them to hand out solid legal advice. They are very knowledgeable.” (VNS91)

“They have more knowledge about animals and animal nutrition than anyone else.” (VNS279)

### Perceived relevance of, and confidence in, nutrition-related patient care and pet caregiver advice

Over three quarters of students (77%, n = 178) believed that diet and nutrition should be evaluated and discussed at every veterinary visit (p = 0.002). The use of evidence-based information by pet caregivers in dietary decision making (93%, n = 213) and nutritional education (98%, n = 226) was considered important to most students which, at this foundation stage of study, is encouraging. However, it is unsurprising that only half of VS (52%, n = 52) and VNS (54%, n = 86) felt sufficiently knowledgeable to independently access current, reliable and evidence-based nutritional information.

### Nutrition-related information and experience

**Fig 7:**
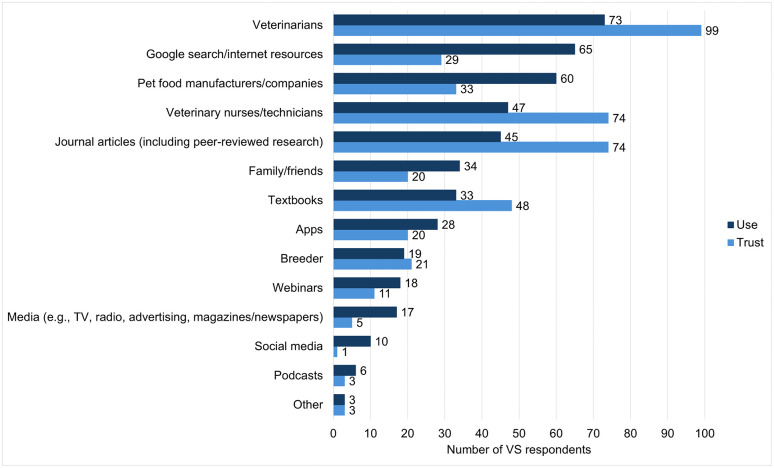
Sources of information that veterinary students (VS) use and/or trust to learn more about small animal nutrition.

**Fig 8:**
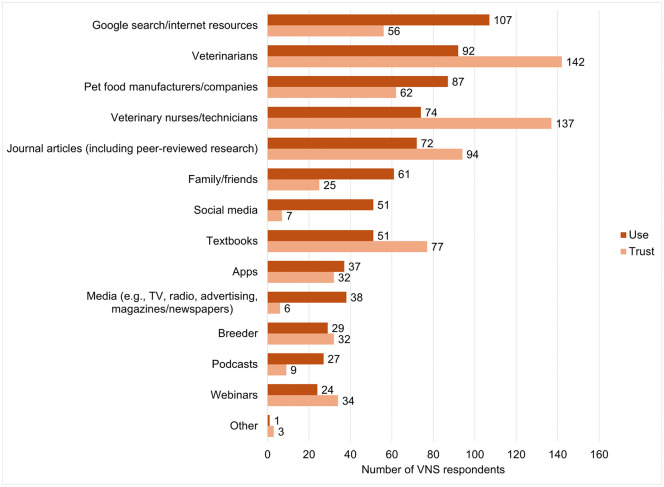
Sources of information that veterinary nurse students (VNS) use and/or trust to learn more about small animal nutrition.

This investigation revealed that use of information source does not necessarily correspond to the level of trust assigned to it. Both student groups trusted expert sources of nutrition information, favouring veterinarians, veterinary nurses/technicians and journal articles, including peer-reviewed research (Figs 7 and 8). Yet, the internet was identified as the most, and second most used resource by VNS and VS, respectively and, overall, use of non-expert information sources was generally far higher than the associated level of trust. For example, 22% of students use social media but only 3% trust it and 34% discuss pet nutrition with family and friends but their viewpoint is trusted by only 16%.

**Fig 9:**
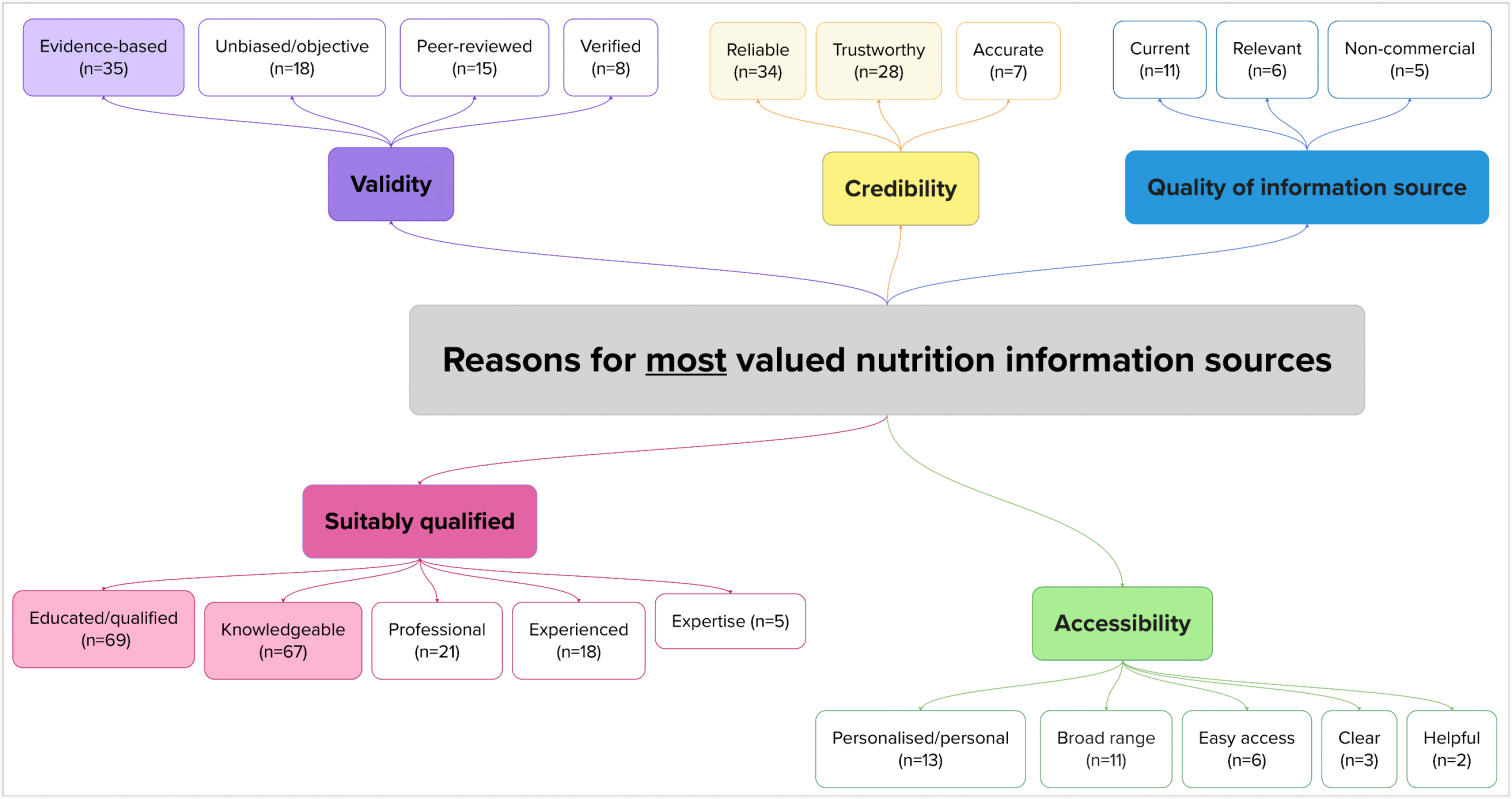
Themes and categories of participants’ (n = 279) reasons for their most valued small animal nutrition information sources. (Created using Mural (https://www.mural.co)).

When asked to rank the three most valued information sources, students cited veterinarians (92%, n = 257), veterinary nurses/technicians (71%, n = 197) and journal articles, including peer reviewed research (46%, n = 128). Thematic analysis of respondents’ reasons for this selection revealed several categories (Fig 9). The top five were educated/qualified (n = 69), knowledgeable (n = 67), evidence-based (n = 35), reliable (n = 34) and trustworthy (n = 28). From these, five overarching themes emerged: validity, credibility, quality of information source, suitably qualified and accessibility. Respondents’ provision of free text comments included:

“Peer reviewed journals usually provide scientific evidence and are up to date. Veterinary professionals have a wide knowledge and interest in animal nutrition.” (VS38)

“Most unbiased sources” (VNS47)

“I feel like vets and vet nurses must have the knowledge prior their graduation and so I trust their opinion.” (VNS62)

“They gain nothing from being honest and want the best for your animal.” (VS66)

“Vets/veterinary nurses are knowledgeable and have studied animals to know their needs. Journal articles can offer more information and as they are peer reviewed it gives a sense of trust.” (VS70)

“I have confidence in the education of the people caring for my animal’s health. If a journal has been peer-reviewed and published it is normally safe to assume it holds an up to date understanding of a matter.” (VS71)

“Have more relevant information and can be sure they are doing it for the welfare of the animal not for sales or views” (VNS163)

**Fig 10:**
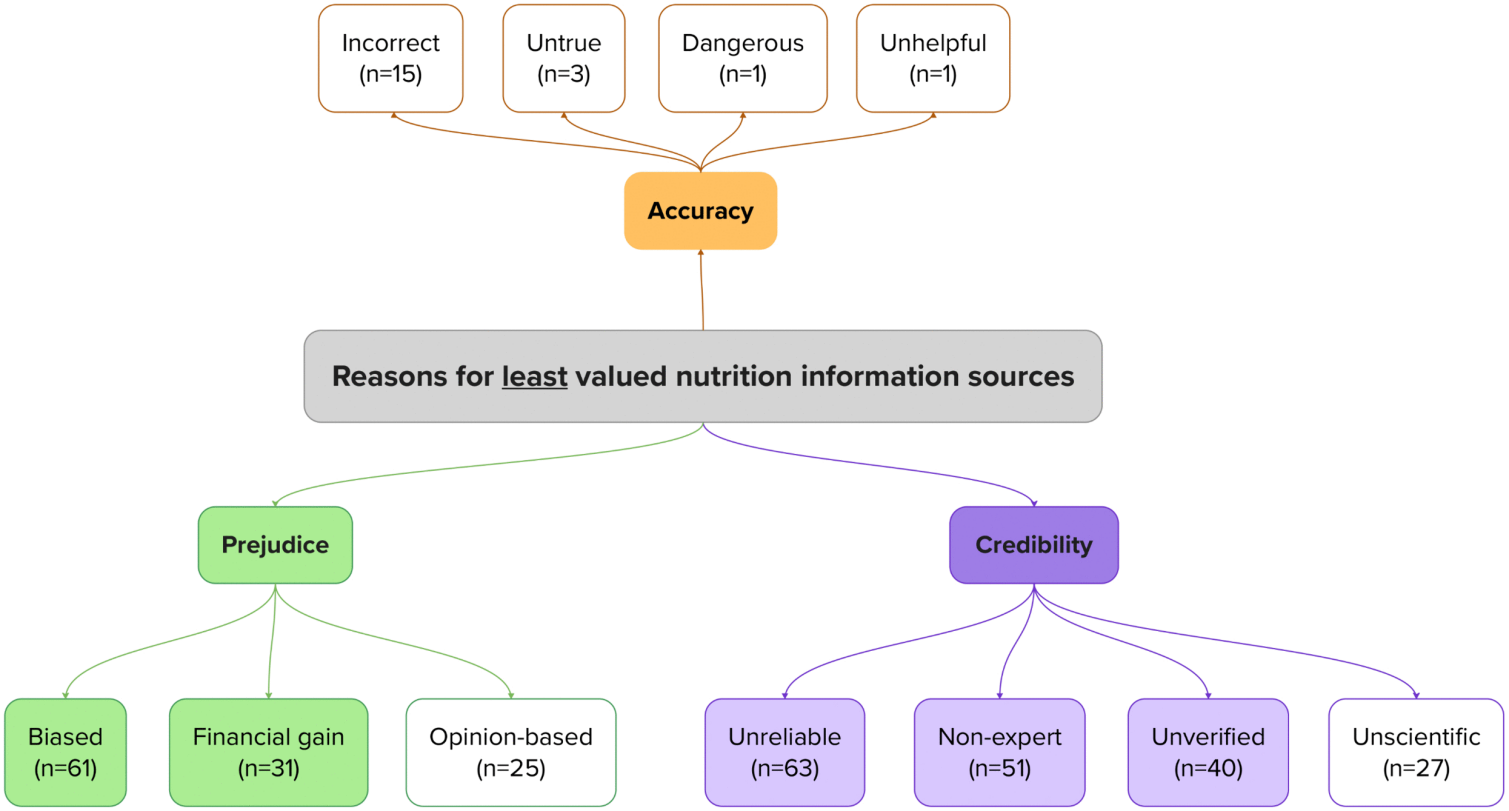
Themes and categories of participants’ (n = 279) reasons for their least valued small animal nutrition information sources. (Created using Mural (https://www.mural.co)).

The three least valued information sources were media (68%, n = 190), social media (65%, n = 180) and family and friends (37%, n = 102). In this context, media refers to a broad range of communication channels including print, broadcast, visual, marketing and news media. It does not include digital or social media. Respondents’ reasons for this selection were described and thematic analysis revealed several categories (Fig 10). The top five were unreliable (n = 63), biased (n = 61), non-expert (n = 51), unverified (n = 40) and financial gain (n = 31). From these, three overarching themes emerged; accuracy, prejudice and credibility. Respondents’ free text comments included:

“A lot of things online are biased and often family and friends aren’t that knowledgeable.” (VS33)

“There’s too much information out there, most of it is subjective and probably not true.” (VNS129)

“They just can be really click bait and trendy and not have the actually best nutritional advice or your animals best nutritional outcomes at heart.” (VS20)

“Wouldn’t trust my family or friends unless in the veterinary background because they are just as knowledgeable as me which is not very knowledgeable.” (VNS63)

“You never know who’s saying what and whether they are promoting things for the right reasons.” (VS22)

“Family and friends, social media and google could not have true facts about proper nutrition, they might have some tips from experiences but some are not trusted sources (google and social media).” (VNS74)

“Family/ friends may go off anecdotal evidence, which can be wrong or biased. Media and social media holds an inherent biased and can exaggerate for the sake of sensationalism.” (VS71)

“Family and friends may not have experience in nutrition when it comes to deciding for an individual pet or species, what diet is suitable for them. And social media and television will always advertise their products even if they aren’t suitable for some pets and could do more harm than good.” (VNS148)

**Fig 11:**
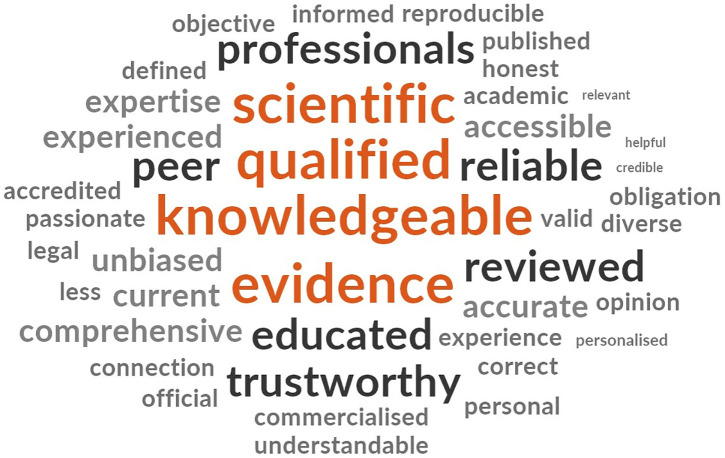
A word cloud depicting the keywords that featured in respondents’ rationale for the selection of most valued nutrition-related information sources. The larger the word, the greater its frequency. Created using NVivo 14 Windows.

**Fig 12:**
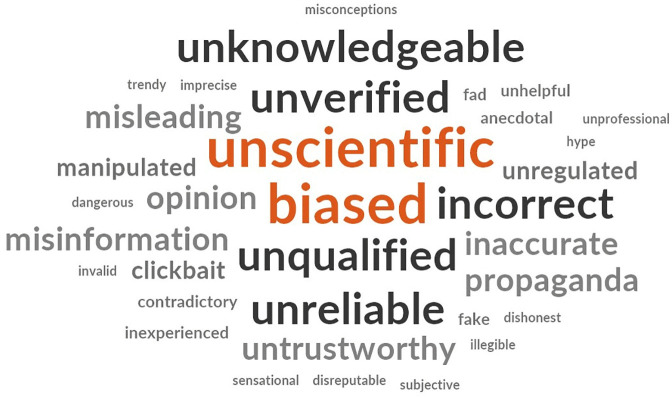
A word cloud depicting the keywords that featured in respondents’ rationale for the selection of least valued nutrition-related information sources. The larger the word, the greater its frequency. Created using NVivo 14 Windows.

Respondents’ comments and rationale for the selection of most and least valued nutrition-related information sources are visually summarised using word clouds (Figs 11 and 12). Commonly repeated words for the reasons for their most valued nutrition-information sources were “knowledgeable” (frequency (f) = 75), “qualified” (f = 37), “evidence” (f = 35), scientific (f = 35), and “educated” (f = 34). When asked for the reasons for their least valued sources of nutrition information, most cited words were “biased” (f = 72), “unscientific” (f = 37), “unqualified” (f = 33), “unreliable” (f = 33) and “unverified” (f = 32).

Open text comments further revealed the incongruity between students’ trust in, and use of, information sources:

“Wrong information can easily be spread in these ways, but [I] would be happy to research suggestions from these sources.” (VS101)

“Vets have an extended knowledge of nutrition. Journal articles have a lot of research going into them, so I tend to trust them, and I use Google if I want to double check something I’ve read.” (VNS58)

“Textbooks and Vets probably have the most information and scientific basis, however I also trust the experience of my friends and family.” (VS68)

“[I] Wouldn’t value internet resources (although I said previously that I trusted them) because many unqualified people can write anything on there which may be ill-advised for nutrition, so it depends on the source of information and who wrote it.” (VNS63)

According to Cronbach’s alpha, the reliability of the questionnaire was good, with alpha = 0.87 (95% confidence interval 0.84–0.89)

## Discussion

The principal objective of the present study was to establish the sources of nutrition information used and trusted by first year (foundation phase) student veterinary professionals. Results highlighted several key findings, including the disconnect between popularity of information sources and perceived trustworthiness.

According to recent evidence, over 80% of veterinarians own at least one pet, with over half having responsibility for multiple household pets [74,75]. Dogs were identified as the most popular pet, followed by cats, fish, birds and small mammals. In the present study, student veterinary professionals’ pet caregiver status paralleled their qualified counterparts, with most having responsibility for the nutritional care of one or more pets, of which dogs, cats and small mammals were most popular. Students’ beliefs surrounding the status of their pet were also comparable to those reported among the general population of pet caregivers. Globally, most caregivers consider their pets to be family members and have a close relationship with their companion animals [10,75]. This was the same for most student veterinary professionals surveyed in the present study.

A strong bond between caregiver and pet is associated with a higher level of expected care than those displaying weaker bonds [76,77]. Several factors, including convenience, trustworthiness and the human companion animal bond, are considered to influence and affect pet caregivers’ choice of information source and information search behaviour [19]. Students’ information seeking behaviour were similar to those of pet caregivers in the general population. The veterinary consultation is identified as a foremost client contact point for nutritional discussion [41] and the veterinary healthcare team are trusted advisors, providing guidance in making informed decisions about preventative care, including diet and nutrition [31,48,78–81]. Similarly, students in the present study considered veterinarians and veterinary nurses to be leading authorities on pet nutrition and placed the highest level of trust in reputable sources of information provided by veterinary professionals and journal articles, including peer-reviewed literature. Yet, similar to Kamleh et al. [48] depending on the species, a minority of student pet caregivers indicated that they discussed nutrition with a veterinary professional. Like veterinarians, veterinary nurses in the UK and Ireland are expected to be competent in a range of nutrition-related skills and competencies by the time of qualification [52–55], yet veterinarians were perceived to have greater knowledge about pet nutrition than veterinary nurses. This may indicate a lack of experience, as observed in a recent survey of the veterinary nursing profession [82]. Over half (54%, n = 1265) of veterinary nurse respondents were involved in nutrition-related nursing clinics. Yet provision of nutritional advice was a less regular task for veterinary nurse respondents, with only a quarter (25%, n = 704) providing nutritional advice/counselling once per working week or less and only just over half (52%, n = 1423) giving weight management advice in the preceding year. Interestingly, in the present study, the nutrition knowledge of qualified veterinary nurses was rated higher by VNS than VS, potentially due to greater awareness and appreciation of the training involved.

Alongside veterinarians, Google search and internet resources were also identified as commonly used information sources by students in the present study, results comparable to pet caregivers based in Canada [35] and in New Zealand where veterinarians and the internet were revealed to be preferred sources of pet-related information by a respective 72% and 51% of pet caregivers [10]. Such findings also mirror those of a global study of over 20,000 dog and cat caregivers, for whom veterinarians had a notable impact on pet food selection, yet family, friends, social media and the internet were cited as most influential information sources [83]. With continued expansion of the internet and an increasing percentage of the global population now connected to the World Wide Web, there is a large abundance of online information at users’ disposal and its influence is growing. The availability of online information about pet health and nutrition is substantial and can be overwhelming. Yet many caregivers consider the internet a favoured [16,84] or complementary [15,17,20,21,26,36,85,86] source of information to the veterinary healthcare team, with pet nutrition and diet one of the most frequently searched topics by pet caregivers [16,17,21].

Whilst many survey respondents in the present study favoured the internet as an information source, this could potentially include reference to reputable websites as well as those that are not. As observed by Nielson et al. [47], given students’ relatively high reliance on peer-reviewed sources, it is possible that they also demonstrated greater discernment when navigating the internet and evaluating online content, but such analysis was beyond the scope of this study. Just over half of respondents felt sufficiently knowledgeable to access reputable, credible and evidence-based nutrition information sources, but this may not be an accurate self-perception, particularly in the foundation stage of study when students have limited information evaluation skills. As identified by Freeman et al. [87] and Gray et al. [88], while young people are aware of the varying quality of online health information, they lack the skills and ability to appraise its credibility. Furthermore, Gray et al. [88] identified the challenges experienced by young people in locating, evaluating and using online health information.

Overall, students’ use of non-expert information sources was generally far higher than the associated level of trust in it. For example, media, social media, friends and family were considered least valuable, yet respondents’ use was considerably more than their associated trust. Despite providing quick and anonymous access to extensive information [16], online nutrition-related information is frequently inaccurate and of poor quality, with little gatekeeping, putting consumers who seek guidance online at greater risk of being misinformed [89,90]. Furthermore, the views of social media ‘influencers’, friends and family are subject to bias and influenced by personal circumstances, ‘gut feelings’, cultural identity, past experiences, dietary predilections, cost and perceptions surrounding quality [14,89,91,92]. Students in the present study demonstrated scepticism towards information sources, including breeders and pet manufacturers, who were perceived to be benefitting from financial gain and prioritising this over animal welfare.

The disconnect between source popularity and perceived trustworthiness observed in the present study is not unique to student veterinary professionals and mirrors the literature amongst nutrition healthcare. Pavlović et al [93] explored university students’ nutrition-related information-seeking behaviours and revealed similar findings whereby expert sources of information are highly trusted but infrequently used. In contrast, non-expert information sources (the internet, media, family and friends) were the most used information source on nutrition, yet trust was relatively low and variable according to site. This is in line with Ruani et al [94] who found that frequent information seeking from inferior sources did not reflect the level of trust ascribed to it; non-expert sources were commonly consulted, despite highest trust being placed in expert sources.

Evidence has revealed the pronounced influence that trustworthiness of information sources has on the likelihood of dietary modification, with reliance on more trustworthy sources more likely to translate into effective diet change [94]. Student veterinary professionals’ perceptions of, and trust in, nutritional information are shaped not only by academic learning but also by real-world application. Many of the students surveyed are also pet caregivers and clients for whom personal experience, perceptions, marketing, trends, and misinformation can influence and inform their current dietary decision-making. Crucially, this is also likely to shape their future communication with clients and application of nutrition advice as qualified veterinarians and veterinary nurses. Globally, trust and confidence in information on pet nutrition and healthcare obtained from veterinary professionals is reportedly high [14,26,80,95] and pet caregivers expect veterinarians to provide guidance about online content [16,26,96]. Fostering students’ trust in evidence-based nutrition, development of information literacy and ability to effectively convey credible information is therefore critical, not only for their own animals, but for the clients and patients they will one day serve.

### Clinical implications

The current findings highlight the central role of the veterinary team in guiding evidence-based dietary decision-making. Student agreement that nutrition should be assessed at every veterinary visit demonstrates their awareness of nutrition as a fundamental component of health and welfare. Veterinarians and veterinary nurses were perceived as knowledgeable and trusted sources of nutrition information, yet veterinary nurses were consulted less frequently than veterinarians regarding pet nutrition. This highlights an opportunity to better utilise veterinary nurses for the teaching of student veterinary professionals and enhancement of client education and continuity of care. It further underscores the need for veterinary practices to proactively address nutrition during routine consultations, rather than relying on caregivers to seek advice independently. This is particularly important for exotic pet species. Students’ high reliance on non-expert sources risks misinformation, potentially influencing personal and future professional decision-making. Furthermore, students’ limited confidence in accessing evidence-based nutrition information reinforces the need for relevant undergraduate education, along with appropriate signposting to suitable resources. Collectively, these findings emphasise the importance of a team-based, evidence-led approach to nutrition-related care and education.

### Strengths and limitations of present study

The present study offers much needed insight into the preferred sources of information used and trusted by student veterinary professionals to learn more about small animal nutrition. It is unique in its ability to compare the views of veterinary and veterinary nursing students, enabling a direct comparison of each professional group, and the findings have potential implications for interprofessional education and practice.

A notable strength of the current study is its consideration of student veterinary nurses, whose perspectives are underrepresented in literature. Unlike veterinary students, student veterinary nurses in the UK and Ireland are required to demonstrate competence in a range of clinical skills, including nutrition-related tasks, prior to qualification. Beliefs and habits formed during training are more likely to continue into professional life. Early establishment of used and trusted nutrition sources is therefore fundamental to reinforcing critical appraisal skills and promoting the use of evidence-based information. Qualified veterinary nurses are frontline veterinary professionals who play a vital role in educating clients about nutrition, making dietary recommendations, implementing nutritional plans and monitoring patient outcomes. Advocating an appropriate diet has a measurable impact on animal and human health and well-being and is reliant on trusting, applying and communicating sound nutrition information.

Further strengths include pilot testing of the survey which enhanced the study’s internal validity. Whilst the study failed to distinguish between the types of internet sites and online resources utilised by student veterinary professionals, it has revealed their perceived value, paving the way for future analysis of students’ online search strategies.

The findings of the study apply to the study participants. Due to the relatively low sample size, and consequently the margin of error associated with extrapolation, the findings may not be generalisable to the entire population of veterinary and veterinary nurse students. Future research that obtains a truly representative sample (rather than a convenience sample) would be required in order to generalise findings to the wider population.

## Conclusion

This investigation highlighted several key findings, including the fact that frequent utilisation of an information source is not necessarily a reliable predictor of its perceived trustworthiness. Findings from this study will guide future research and intervention approaches aimed at enhancing the information literacy skills of student veterinary professionals in the foundation phase of study. It could also encourage more robust curricula and evidence-based training in nutrition, in turn increasing the confidence of newly qualified veterinary professionals to support pet caregivers in making evidence-based dietary decisions and signposting to appropriate information sources and reputable websites.

Given the rising popularity of unconventional and alternative diets and the availability of unsubstantiated information, it will be interesting to ascertain if improvements are determined at later stages of the education programme for veterinary and veterinary nursing students. Future research could compare the knowledge and attitudes of foundation phase (first-year) and professional phase (penultimate year) students, as well as qualified veterinarians and veterinary nurses.

## Supporting information

S1 File Questionnaire completed by student veterinarians. (DOCX)

S2 File Questionnaire completed by student veterinary nurses. (DOCX)
