# Supplementary material for: A survey of UK and Irish veterinary and veterinary nursing Students’ engagement with small animal nutrition information
Source: PLoS One. 2026 Jul 16;21(7):e0351963. doi: 10.1371/journal.pone.0351963 (PMC13374976; doi:10.1371/journal.pone.0351963)
Supplement: S2 File — (DOCX) [file pone.0351963.s002.docx]

Section 1: Demographics

1. Please identify your age in years

- Less than 20
- 21 – 30
- 31 – 40
- Over 40

1. What gender do you identify with?

- Female
- Male
- Other (please specify) __________________
- Prefer not to answer

1. Which College/University are you studying at?

- Aberystwyth University
- Atlantic Technological University
- Coleg Gwent (University of South Wales)
- College of Animal Welfare (Middlesex University)
- Dundalk Institute of Technology Harper Adams University
- Hartpury University
- Munster Technological University
- Myerscough College (University of Central Lancashire)
- Nottingham Trent University
- Reaseheath College (University of Chester)
- Royal Veterinary College (University of London)
- Scotland's Rural University College (University of Glasgow)
- Sparsholt College (University of Portsmouth)
- Technological University of the Shannon - TUS
- University Centre Askham Bryan
- University College Dublin
- University of Bristol
- Warwickshire College (Coventry University)
- Wrexham Glyndwr University

1. Please state the postcode of your permanent address (i.e. your family home rather than your temporary university-based residence)

Section 2: Pet ownership

1. Have you got, or ever had, responsibility for the food selection and feeding management of any of the following pets? Please select all that apply.

- Yes, dog(s) (continue to Q6)
- Yes, cat(s) (continue to Q6)
- Yes, rabbit(s) (continue to Q6)
- Yes, small pet mammal(s), including rodents (continue to Q6)
- Yes, reptile(s) (continue to Q6)
- Yes, bird(s) (continue to Q6)
- Yes, fish (continue to Q6)
- Yes, amphibians (continue to Q6)
- Yes, invertebrates, including arachnids and insects (continue to Q6)
- No (continue to Q12)

**Please answer questions 5 – 10 only in relation to the species of pets you selected in the previous question.**

1. Would you consider the pet(s) to be a member of your family?

|  | Yes | No | Not sure |
| --- | --- | --- | --- |
| Dogs |  |  |  |
| Cats |  |  |  |
| Rabbits |  |  |  |
| Small pet mammals, including rodents |  |  |  |
| Reptiles |  |  |  |
| Birds |  |  |  |
| Fish |  |  |  |
| Amphibians |  |  |  |
| Invertebrates, including arachnids and insects |  |  |  |

1. How knowledgeable do you feel regarding the nutrition of the pet(s)?

|  | 1 – No knowledge | 2 – Not very | 3 – Neutral | 4 – Somewhat | 5 – Very knowledgeable |
| --- | --- | --- | --- | --- | --- |
| Dogs |  |  |  |  |  |
| Cats |  |  |  |  |  |
| Rabbits |  |  |  |  |  |
| Small mammals, including rodents |  |  |  |  |  |
| Reptiles |  |  |  |  |  |
| Birds |  |  |  |  |  |
| Fish |  |  |  |  |  |
| Amphibians |  |  |  |  |  |
| Invertebrates, including arachnids and insects |  |  |  |  |  |

1. Have you ever discussed the nutrition of the pet(s) with your **veterinarian**?

|  | Yes (continue to Q9) | No (continue to Q10) | Not sure (continue to Q10) |
| --- | --- | --- | --- |
| Dogs |  |  |  |
| Cats |  |  |  |
| Rabbits |  |  |  |
| Small mammals, including rodents |  |  |  |
| Reptiles |  |  |  |
| Birds |  |  |  |
| Fish |  |  |  |
| Amphibians |  |  |  |
| Invertebrates, including arachnids and insects |  |  |  |

1. How knowledgeable do you feel the **veterinarian** was regarding the nutrition of the pet(s)?

|  | 1 – No knowledge | 2 – Not very | 3 – Neutral | 4 – Somewhat | 5 – Very knowledgeable |
| --- | --- | --- | --- | --- | --- |
| Dogs |  |  |  |  |  |
| Cats |  |  |  |  |  |
| Rabbits |  |  |  |  |  |
| Small mammals, including rodents |  |  |  |  |  |
| Reptiles |  |  |  |  |  |
| Birds |  |  |  |  |  |
| Fish |  |  |  |  |  |
| Amphibians |  |  |  |  |  |
| Invertebrates, including arachnids and insects |  |  |  |  |  |

1. Have you ever discussed the nutrition of the pet(s) with a **veterinary nurse/technician**?

|  | Yes (continue to Q11) | No (continue to Q12) | Not sure (continue to Q12) |
| --- | --- | --- | --- |
| Dogs |  |  |  |
| Cats |  |  |  |
| Rabbits |  |  |  |
| Small mammals, including rodents |  |  |  |
| Reptiles |  |  |  |
| Birds |  |  |  |
| Fish |  |  |  |
| Amphibians |  |  |  |
| Invertebrates, including arachnids and insects |  |  |  |

1. How knowledgeable do you feel the **veterinary nurse/technician** was regarding the nutrition of these pet(s)?

|  | 1 – No knowledge | 2 – Not very | 3 – Neutral | 4 – Somewhat | 5 – Very knowledgeable |
| --- | --- | --- | --- | --- | --- |
| Dogs |  |  |  |  |  |
| Cats |  |  |  |  |  |
| Rabbits |  |  |  |  |  |
| Small mammals, including rodents |  |  |  |  |  |
| Reptiles |  |  |  |  |  |
| Birds |  |  |  |  |  |
| Fish |  |  |  |  |  |
| Amphibians |  |  |  |  |  |
| Invertebrates, including arachnids and insects |  |  |  |  |  |

Section 3: Nutrition-related information and experience

1. Prior to enrolling as a student veterinary nurse, have you completed any professional development or training on the topic of small animal nutrition?

- Yes
- No
- Not sure / can’t remember

1. Which sources of information do you use and/or trust to learn more about **small animal nutrition**? Please select all that apply.

|  | Use | Trust |
| --- | --- | --- |
| Apps |  |  |
| Breeder |  |  |
| Family / Friends |  |  |
| Google search / Internet resources (e.g., online blogs, online articles) |  |  |
| Media (e.g., television, radio, advertising, magazines/newspapers) |  |  |
| Journal articles |  |  |
| Pet food manufacturers |  |  |
| Podcasts |  |  |
| Social media |  |  |
| Textbooks |  |  |
| Veterinarian |  |  |
| Veterinary Nurse/Technician |  |  |
| Webinars |  |  |
| Other (please specify) _______________________________________ |  |  |

1. Which **THREE** sources would you value the **most**?

- Apps
- Breeder
- Family / Friends
- Google search / Internet resources (e.g., online blogs, online articles)
- Media (e.g., television, radio, advertising, magazines/newspapers)
- Journal articles
- Pet food manufacturers
- Podcasts
- Social media
- Textbooks
- Veterinarian
- Veterinary Nurse/Technician
- Webinars
- Other (please specify) ________________________________________

1. Explain the reasons for your choice (optional)

1. Which **THREE** sources would you value the **least**?

- Apps
- Breeder
- Family / Friends
- Google search / Internet resources (e.g., online blogs, online articles)
- Media (e.g., television, radio, advertising, magazines/newspapers)
- Journal articles
- Pet food manufacturers
- Podcasts
- Social media
- Textbooks
- Veterinarian
- Veterinary Nurse/Technician
- Webinars
- Other (please specify) ________________________________________

1. Explain the reasons for your choice (optional)

**Please answer the following questions in relation to the time you have spent working or gaining experience with small animals in a veterinary practice.**

1. Which of the following nutrition tasks have you **observed**? Please select all that apply.

- Clients purchasing pet food
- Assessment of body condition score (BCS)
- Assessment of muscle condition score (MCS)
- Calculation of energy requirements
- Formulation of a nutritional plan
- Obtaining a pet’s dietary history from a client
- Preparation of a diet to be fed to a hospitalised patient
- Placement of a feeding tube
- Administration of food via a bowl
- Administration of food via a feeding tube
- Administration of food via hand or coax feeding
- Administration of food via a syringe
- None of the above
- Not sure / can’t remember

1. Have you **observed** a client discussing their pet’s **diet choice / feeding management** with the following team members? Please select all that apply.

- Yes, with a veterinarian
- Yes, with a veterinary nurse/technician
- Yes, with a receptionist
- No
- Not sure / can’t remember

1. Have you **observed** a client discussing their pet’s **body weight** with the following team members? Please select all that apply.

- Yes, with a veterinarian
- Yes, with a veterinary nurse/technician
- Yes, with a receptionist
- No
- Not sure / can’t remember

1. Have you **observed** a client discussing their pet’s **body condition** with the following team members? Please select all that apply.

- Yes, with a veterinarian
- Yes, with a veterinary nurse/technician
- Yes, with a receptionist
- No
- Not sure / can’t remember

1. Which of the following tasks have you **conducted** or **assisted with**? Please select all that apply.

- Clients purchasing pet food
- Assessment of body condition score (BCS)
- Assessment of muscle condition score (MCS)
- Calculation of energy requirements
- Formulation of a nutritional plan
- Obtaining a pet’s dietary history from a client
- Preparation of a diet to be fed to a hospitalised patient
- Placement of a feeding tube
- Administration of food via a bowl
- Administration of food via a feeding tube
- Administration of food via hand or coax feeding
- Administration of food via a syringe
- None of the above
- Not sure / can’t remember

1. Which of the following species have you helped to **feed** whilst hospitalised in a veterinary practice? Please select all that apply.

- Dogs
- Cats
- Rabbits
- Small mammals, including rodents
- Reptiles
- Birds
- Fish
- Amphibians
- Invertebrates, including arachnids and insects
- Other (please specify) ____
- None of the above
- Not sure / can’t remember

Section 4: Perceived relevance and importance of nutrition-related education

|  | 1 – Strongly Disagree | 2 – Disagree | 3 – Undecided | 4 – Agree | 5 – Strongly agree |
| --- | --- | --- | --- | --- | --- |
| 1. I am interested in small animal nutrition |  |  |  |  |  |
| 1. I enjoy learning about small animal nutrition |  |  |  |  |  |
| 1. I know how to access current, reliable and evidence-based information about small animal nutrition |  |  |  |  |  |
| 1. I am confident that the nutrition training I receive during my degree programme will prepare me for work as a registered veterinary nurse. |  |  |  |  |  |

| **It is important that I develop the knowledge and skills to be able to correctly:** | 1 – Strongly Disagree | 2 – Disagree | 3 – Undecided | 4 – Agree | 5 – Strongly agree |
| --- | --- | --- | --- | --- | --- |
| 1. Perform a clinical examination (including assessment of body condition score) of a small animal |  |  |  |  |  |
| 1. Assess the nutritional status (presence or absence of malnutrition) of a small animal |  |  |  |  |  |
| 1. Educate pet owners on good practice of small animal husbandry and feeding |  |  |  |  |  |
| 1. Educate owners about nutrition appropriate to a pet’s species and life stage |  |  |  |  |  |
| 1. Educate owners about healthy weight management for their pet(s) |  |  |  |  |  |

Section 5: Self-perceived relevance of, and confidence in, nutrition-related patient care and pet owner advice

|  | 1 – Strongly Disagree | 2 – Disagree | 3 – Undecided | 4 – Agree | 5 – Strongly agree |
| --- | --- | --- | --- | --- | --- |
| 1. A pet’s diet and nutrition status should be evaluated and discussed with the owner at every veterinary visit |  |  |  |  |  |
| 1. It is important that owners make evidence-based decisions regarding their pet’s diet and feeding management |  |  |  |  |  |
| 1. It is important that I use evidence-based information when educating pet owners about nutrition |  |  |  |  |  |
| 1. A commercially produced raw diet is healthier than a commercially produced cooked diet. |  |  |  |  |  |
| 1. The risks of raw feeding outweigh the benefits. |  |  |  |  |  |
| 1. 'By-product' ingredients are unsuitable for human consumption and may harm pets. |  |  |  |  |  |

1. Do you believe a vegetarian diet is nutritionally adequate for cats and dogs?
2. Please identify up to **FIVE** potential risks of feeding a raw diet to dogs and cats
3. Which **ONE** of the following statements correctly describes the ideal body condition score for a cat?

- Waist poorly discernible. Ribs not easily palpated. Moderate abdominal fat pad
- Very obvious waist behind ribs. Ribs are visible and no palpable fat.
- Waist and abdominal fat pad distinguishable but not obvious. Ribs palpable.
- The waist is visible behind the ribs. The ribs are palpable with slight fat covering. Abdominal fat pad minimal.

| **I feel confident in my ability to correctly:** | 1 – Strongly Disagree | 2 – Disagree | 3 – Undecided | 4 – Agree | 5 – Strongly agree |
| --- | --- | --- | --- | --- | --- |
| 1. Perform a clinical examination (including assessment of body condition score) of a **dog** and **cat** |  |  |  |  |  |
| 1. Perform a clinical examination (including assessment of body condition score) of an **exotic pet** (including small mammals, birds and reptiles) |  |  |  |  |  |
| 1. Assess the nutritional status of a **dog** and **cat** |  |  |  |  |  |
| 1. Assess the nutritional status of an **exotic pet** (including small mammals, birds and reptiles) |  |  |  |  |  |
| 1. Assess the body weight of a **dog** and **cat** |  |  |  |  |  |
| 1. Assess the body weight of an **exotic pet** (including small mammals, birds and reptiles) |  |  |  |  |  |
| 1. Calculate food requirements of a **dog** and **cat** based on disease and life stage/lifestyle |  |  |  |  |  |
| 1. Determine appropriate feeding and nutrition goals for a patient |  |  |  |  |  |
| 1. Ascertain information about a patient’s normal routine with respect to food and fluid intake |  |  |  |  |  |
| 1. Manage assisted feeding through hand feeding |  |  |  |  |  |
| 1. Manage assisted feeding through oral hydration |  |  |  |  |  |
| 1. Manage and maintain feeding tubes |  |  |  |  |  |
| 1. Advise the owner of a **dog** or **cat** on good practice of husbandry and nutritional needs |  |  |  |  |  |
| 1. Advise the owner of an **exotic pet** (including small mammals, birds and reptiles) on good practice of husbandry and nutritional needs |  |  |  |  |  |
| 1. Advise clients on specialised nutritional diets for their animals |  |  |  |  |  |
| 1. Communicate with the owner of an **overweight pet** about their pet’s body condition |  |  |  |  |  |
| 1. Advise the owner of an overweight **cat** about weight loss strategies |  |  |  |  |  |
| 1. Advise the owner of an overweight **dog** about weight loss strategies |  |  |  |  |  |
| 1. Advise the owner of an overweight **rabbit** about weight loss strategies |  |  |  |  |  |
| 1. Maintain accurate, clear and concise records regarding the nutrition-related advice and recommendations I provide to clients |  |  |  |  |  |
| 1. Maintain accurate, clear and concise records regarding the nutrition-related care I provide to patients |  |  |  |  |  |
