# Supplementary material for: A survey of UK and Irish veterinary and veterinary nursing Students’ engagement with small animal nutrition information
Source: PLoS One. 2026 Jul 16;21(7):e0351963. doi: 10.1371/journal.pone.0351963 (PMC13374976; doi:10.1371/journal.pone.0351963)
Supplement: Attachment — Submitted filename: Response to Reviewers PONE-D-25-21707R1.docx [file pone.0351963.s006.docx]

# Response to reviewers in relation to manuscript ID: PONE-D-25-21707R1

# ‘A Survey of UK and Irish Veterinary and Veterinary Nursing Students’ Engagement with Small Animal Nutrition Information’.

The authors would like to thank the Academic Editor and the reviewers for the time and effort devoted to reviewing our manuscript. We appreciate the constructive comments, and we have revised the manuscript accordingly. Please find below details of how these comments have been addressed. The line numbers are correct according to the track changes version of the revised manuscript.

| **Section** | **Line(s)** | **Comment** | **Action taken** |
| --- | --- | --- | --- |
| Abstract | Line 24 | Use the possessive form of “study” | Word changed to study’s |
| Introduction | Lines 102 to 106 | Paraphrase the following to make meaning clearer: “This manuscript presents results obtained as part of a broader longitudinal two-part project to evaluate and compare the small animal nutrition-related knowledge, attitudes and competence of foundation and professional phase veterinary students (VS) and veterinary nursing students (VNS).” | This has been split into two sentences. |
| Methods | Lines 118 to 120 | Paraphrase the following to make meaning clearer: “The study design and questionnaire was reviewed and given ethics approval by the University of Glasgow, College of Medical, Veterinary & Life Sciences Ethics Committee (reference number 200230001).” | This sentence has been paraphrased. |
|  | Lines 107 to 108 | Authors must add what “Small Animals” mean or constitute in this study. | A definition has been provided. |

| **Section** | **Line(s)** | **Comment** | **Action taken** |
| --- | --- | --- | --- |
| Methods | Lines 26 to 27 and 130 to 131 | Study participants  Authors must clearly explained how they arrived at the sample size. If No formula was employed, they must calculate the power of the study. | Participants were recruited by non-probability, convenience purposive sampling, by email invitation from educational providers. During the design of this study, the authors sought expert advice regarding power analysis from a statistician and Lecturer in Research and Numerical Skills at the University of Glasgow. Given the recruitment of participants using non-probability, convenience purposive sampling, the fact that the sample size would be fixed (based on the number of participants recruited), and the fact that specific comparisons were not being made/tested, power calculations were deemed inappropriate.  From the authors’ knowledge and experience, power analysis can help inform sample size decisions for a future study that has not yet taken place. Using it to interpret the results of an already completed study is usually considered inappropriate due to the high potential for logically invalid and practically misleading results. Expert advice has confirmed this viewpoint, and the authors have been warned against the use of post-hoc power analysis for this study. Data obtained in the present study could be used to inform power calculations for future studies which test specific hypotheses/make specific comparisons. |
|  | Lines 161 to 168 | Questionnaire design  What was the internal consistency and reliability score of the Questionnaire? State the Cronbach's alpha score. | The questionnaires were piloted by a range of individuals, including an academic and expert in research design. The mixed-methods nature of the study meant that each individual contributed unique subjective information rather than objective information that could be assessed using any test-retest framework. The authors have sought expert advice, as noted immediately above, and been warned against the use of Cronbach's alpha as an accurate estimate of reliability for this study due to a lack of measurement of the same underlying construct and the common misconception when using Cronbach’s alpha that a high value would be indicative of homogeneity between items. <https://en.wikipedia.org/wiki/Cronbach%27s_alpha#Common_misconceptions> |

| **Section** | **Line(s)** | **Comment** | **Action taken** |
| --- | --- | --- | --- |
| Methods | Lines 174 to 175 | Data collection  What mechanism was put in place to ensure that participants don’t submit two or more responses? | Survey settings ensured that duplicate respondents were prevented from taking the survey multiple times. This has been clarified in the manuscript. |
|  | Lines 180 to 181 | Data analysis  State the version of the Microsoft Excel used for the analyses. | This detail has now been added. The version used was Microsoft® Excel® for Microsoft 365 MSO (Version 2405). |
| Results | Lines 217 to 222 | Pet ownership  Poor mathematical presentation of results. P-values such as (p=<.015), (p=<.021), etc must be presented professionally. | The necessary amendments have been made to ensure professional presentation of p-values. |
| Discussion | Lines 478 to 494 | The Discussion lacks clinical implication of results. Authors must include Clinical Implications. | The Discussion has been revised to include a dedicated paragraph outlining the clinical implication of results. |
| Acknowledgements | Lines 548 to 550 | Please remove any funding-related text from the manuscript and let us know how you would like to update your Funding Statement. Currently, your Funding Statement reads as follows: “This paper has been produced as part of the first author’s (RL) doctoral degree which is being funded by the University of Glasgow, Small Animal Hospital Fund (The Ronald Miller Foundation). The funders played no role in the study design, data collection and analysis, decision to publish, or preparation of the manuscript.” | Funding-related text has been removed from the Acknowledgements section. The authors do not wish to make any changes to the Funding Statement. |

| **Section** | **Line(s)** | **Comment** | **Action taken** |
| --- | --- | --- | --- |
| Acknowledgements | Lines 541 to 546 | If there are ethical or legal restrictions on sharing a de-identified data set, please explain them in detail (e.g., data contain potentially identifying or sensitive patient information, data are owned by a third-party organization, etc.) and who has imposed them (e.g., a Research Ethics Committee or Institutional Review Board, etc.). Please also provide contact information for a data access committee, ethics committee, or other institutional body to which data requests may be sent. | Ethical approval was granted by the University of Glasgow, College of Medical, Veterinary & Life Sciences Ethics Committee based on the condition that other researchers can have access to this data only if they have scientific and ethical approval. A user license has been created by the University of Glasgow Contracts Team, which captures these restrictions and enables data to be made available under controlled access. Further information, including requests for access, can be made through the University of Glasgow Enlighten publications repository at <https://doi.org/10.5525/gla.researchdata.1951>. This site will go live following acceptance of the manuscript for publication. |
