# Supplementary material for: A survey of UK and Irish veterinary and veterinary nursing Students’ engagement with small animal nutrition information
Source: PLoS One. 2026 Jul 16;21(7):e0351963. doi: 10.1371/journal.pone.0351963 (PMC13374976; doi:10.1371/journal.pone.0351963)
Supplement: Attachment — Submitted filename: Response to reviewers PONE-D-25-21707.docx [file pone.0351963.s005.docx]

Response to reviewers in relation to manuscript ID: PONE-D-25-21707

"Used without Trust: Student Veterinary Professionals’ Engagement with Small Animal Nutrition Information."

The authors would like to thank the reviewer and academic editor for their comments regarding this manuscript, and for their specific feedback to help strengthen the publication. Please find below details of how these comments have been addressed.

Reviewer 1:

| **Reviewer’s comment** | **Authors’ response** | **Location of updated information in track changes manuscript** | |
| --- | --- | --- | --- |
|  |  | **Page** | **Line(s)** |
| Title: | | | |
| I found this misleading given that it is in reference to “small animal nutrition information”, implying all small animal nutrition information…that which might be trusted and untrusted. Suggest dropping the “used without trust” part of the title. Also, adding in the geographical locations of the study would be helpful, especially to compare with very similar studies conducted elsewhere (e.g. Canada and the United States). | The title has been changed to, ‘A Survey of UK and Irish Veterinary and Veterinary Nursing Students’ Engagement with Small Animal Nutrition Information’. | 1 | 1-3 |
| Introduction: | | | |
| Line 47: is there a reference to support the “key trend”? Also, the opening sentence is very long and cumbersome – suggest divide into two separate sentences to make the points clearer to follow. | A supporting reference has been added.  The opening sentence has been divided into two separate sentences. | 3 | 48-50 |
| Line 50 & 51: I don’t understand how “greater nutritional variety” is “a veterinary breakthrough for pets” – how so? And why? There are other things that are veterinary breakthroughs. | This sentence has been edited to reflect the important role that nutrition-related support plays in disease prevention and management. | 3 | 50-52 |
| Line 60: I don’t think pet caregivers have a low desire to access reliable and credible information, in fact quite the opposite…I think they do desire such information, and reliable access is the issue. | Reference to ‘desire’ has been removed. | 3 | 62 |
| Method: | | | |
| Questionnaire design: how was the larger questionnaire designed? | Details have been added to explain how the larger questionnaire was designed. A copy of the two versions of the questionnaire (VS and VNS) has also been included as a supplementary file. | 6-7 | 140-147 |
| How did you decide on what questions to include in the questionnaire design? | Information on questionnaire design has been included to clarify this point. | 7 | 151-154 |
| The questions that were used for reporting purposes for this manuscript are vague. While there was mention made of the sections in the questionnaire (sections a-e), there was no detail provided on what the questions were. In fact the methodology section alluded to the idea that there was a larger questionnaire and a sub-set of responses were included in the present manuscript. Please provide detail on the actual questions that were asked and precisely which questions were included for this manuscript. | This is correct – the focus of the current study was to identify the sources of nutrition information used and trusted by first year student veterinary professionals. As such, a relevant subset of responses have been presented in the current manuscript. A copy of the questionnaire used for each student group has been included as supplementary files. The specific questions included for this manuscript have also been made explicit. | 7 | 147-157 |
| Line 190: it is not clear if the participants answered the questions in relation to one animal only or for all the animals that were under their care? Please elaborate how this was handled in the Methods section. | This sentence has been removed and a clearer explanation has been provided within the Methods section. Questions were answered if relevant to the participant and, where applicable, responses reflected the species of pet owned or cared for. | 7 | 155-157 |
| Results: | | | |
| Line 243 & 244 and the paragraph that follows: it is not surprising that first year VS and VNS did not feel sufficient knowable to access evidenced based information themselves since they are likely at the very early stages of learning how to do this. The comments made here should be mitigated with the understanding that these students are an early stage of learning. | Comments now acknowledge students' early stage of education. | 11 | 262-263 |
| Related to the comments above for the Methodology section, it is unclear what results related to which questions. Can the authors provide the precise questions that were answered by the VS and VNS in the Methodology section and then subsequently present the data that was collected from these questions in the Results sections? | The precise questions have been referenced within the Methods section. The data collected from these questions has been presented in the results section under subheadings that align to the relevant section of the questionnaire (a to e). | 7 | 151-157 |
| Using the same wording for both the questions in the Methods and sub-headings in the Results section will help the reader to match up questions with outcome data. Currently, there are two sub-headings used in the Results section that are vaguely related to the mentioned sections a-e in the questionnaire but not entirely clear as to how these sections relate to the questions that were presented to students. | Within the Results, five subheadings have been added which align with the five sections (a-e) of the questionnaire and are listed within the Methods section. This makes it easier for readers to match up questions with outcome data and to also identify in which section of the questionnaire, the questions were included. | 9-12 | 192-268 |
| Discussion: | | | |
| Lines 407 – 410: I may have missed this aspect in the Results section, I did not see data that related to participants’ confidence in their ability to decipher information? The comments above for the Methods & Results section to make apparent what questions were asked and what their outcomes were, may help with this. | The sentence ‘*Respondents had confidence in their ability to decipher information and identify the most reputable and credible information sources*…’ has been changed to ‘*Just over half of respondents felt sufficiently knowledgeable to access reputable, credible and evidence-based nutrition information sources*…’. The corresponding results are reported on lines 263-266 of the Results section on p11-12. | 18 | 432-434 |
| Overall comment for Discussion section: it seemed that most of the outcomes from this study were comparable to similar studies previously conducted for veterinary students and sources of nutrition information and perceptions of trust. The one unique aspect about this study is the inclusion of vet nursing students – I think the authors could expand the overall relevance and future impact of VNS perceptions and trust in information. | Thank you for highlighting this important omission. We have now acknowledged the inclusion and consideration of student veterinary nurses as a notable strength of the study. | 21 | 493-504 |
| One important aspect that was missed in the Discussion is the fact that VS & VNS are also clients. They themselves own and care for animals making their perceptions and trust in nutrition information even more impactful for when they themselves are servicing clients as vet nurses and veterinarians. I would encourage the authors to consider discussing this aspect and relating the impact of this to the future roles of the participants. | Thank you for making this valuable observation. We have now edited the discussion accordingly. | 20 | 469-484 |

Academic Editor:

| **Academic Editor’s comment** | **Authors’ response** | **Location of updated information** | |
| --- | --- | --- | --- |
|  |  | **Page** | **Line(s)** |
| The manuscript requires extensive revisions that fundamentally alter its structure, clarity, and focus. Given the depth of changes needed—particularly in methodology transparency, results presentation, and discussion framing. | The authors believe the required revisions to be relatively minor and presentational in nature. We have done our best to address the comments. | N/A | N/A |
| 1-The title "Used without Trust" is misleading and should be revised to reflect the geographical scope and focus on information-seeking behavior. | The title has been changed to, ‘A Survey of UK and Irish Veterinary and Veterinary Nursing Students’ Engagement with Small Animal Nutrition Information’. | 1 | 1-3 |
| 2-Line 47: Clarify "key trend" with a reference. | A supporting reference has been added. | 3 | 48-50 |
| 3-Lines 50–51: Justify "veterinary breakthrough" claim. | This sentence has been edited to reflect the important role that nutrition-related support plays in disease prevention and management. | 3 | 50-52 |
| 4-Line 60: Rephrase "low desire" to reflect caregivers’ actual motivations. | ‘Low desire’ was the term used by the reviewer. Reference to ‘desire’ has been deleted. | 3 | 62 |
| 5-Line 47: Add references. | A supporting reference has been added. | 3 | 48-50 |
| 6-Methodology: Lack of details on questionnaire design and specific questions analyzed. | This information has been added to the Methods section. | 6-7 | 140-157 |
| 7-Result: Misalignment between Methods and Results subsections, and overinterpretation of first-year students’ lack of confidence. | Within the Results, five subheadings have been added which align with the five sections (a-e) of the questionnaire and are listed within the Methods section. This makes it easier for readers to match up questions with outcome data and to also identify in which section of the questionnaire, the questions were included. | 9-12 | 192-268 |
