# Supplementary material for: A survey of UK and Irish veterinary and veterinary nursing Students’ engagement with small animal nutrition information
Source: PLoS One. 2026 Jul 16;21(7):e0351963. doi: 10.1371/journal.pone.0351963 (PMC13374976; doi:10.1371/journal.pone.0351963)
Supplement: Attachment — Submitted filename: Comments to AuthorsPONE.docx [file pone.0351963.s004.docx]

Abstract

Line 24 – Use the possessive form of “study”

Introduction

Lines 102 to 105: Paraphrase the following to make meaning clearer: “This manuscript presents results obtained as part of a broader longitudinal two-part project to evaluate and compare the small animal nutrition-related knowledge, attitudes and competence of foundation and professional phase veterinary students (VS) and veterinary nursing students (VNS).”

Methods

Lines 114 to 117: Paraphrase the following to make meaning clearer: “ The study design and questionnaire was reviewed and given ethics approval by the University of Glasgow, College of Medical, Veterinary & Life Sciences Ethics Committee (reference number 200230001).”

Authors must add what “Small Animals” mean or constitute in this study.

Study participants

Authors must clearly explained how they arrived at the sample size. If No formula was employed, they must calculate the power of the study.

Questionnaire design

What was the internal consistency and reliability score of the Questionnaire? State the Cronbach's alpha score.

Data collection

What mechanism was put in place to ensure that participants don’t submit two or more responses?

Data analysis

State the version of the Microsoft Excel used for the analyses.

Results

Pet ownership

Poor mathematical presentation of results. P-values such as (p=<.015), (p=<.021), etc must be presented professionally.

Discussion

The Discussion lacks clinical implication of results. Authors must include Clinical Implications.
