# Supplementary material for: A survey of UK and Irish veterinary and veterinary nursing Students’ engagement with small animal nutrition information
Source: PLoS One. 2026 Jul 16;21(7):e0351963. doi: 10.1371/journal.pone.0351963 (PMC13374976; doi:10.1371/journal.pone.0351963)
Supplement: Attachment — Submitted filename: Response to Reviewers PONE-D-25-21707R2.docx [file pone.0351963.s008.docx]

# Response to reviewers in relation to manuscript ID: PONE-D-25-21707R2

# ‘A Survey of UK and Irish Veterinary and Veterinary Nursing Students’ Engagement with Small Animal Nutrition Information’.

The authors would like to thank the Academic Editor and the reviewers for the time and effort devoted to reviewing our manuscript. We appreciate the constructive comments, and we have revised the manuscript accordingly. Please find below details of how these comments have been addressed. The line numbers are correct according to the marked-up version of the revised manuscript.

| **Section** | **Line(s)** | **Comment** | **Action taken** |
| --- | --- | --- | --- |
| Introduction | 154-156 | Move the following statement to the Methods section “For the purposes of this study, small animal was defined as companion animals or pets, kept for domestic purposes rather than for food production.” | The definition of “small animal” has been moved from the Introduction (lines 105-107) to the Methods section (154-156). |
| Methods | 122-123 | Write full form of “RCVS” | This acronym has been defined in full. |
| Study participants | 127-133 | Please state the population size of both VS and VNS from which you recruited 135 veterinary students (VS) and 186 veterinary nursing students. | This detail has been supplied. |
|  | 133-135 | Scientifically justify why the sample size is good to make general conclusions about the population. If no formula was employed, the authors must calculate the power of the study. | The margin of error has been calculated for the number of responses based on the maximum potential population. |
| Questionnaire design | 177-178 | This sentence has already been stated above. Delete it. “An online pre-test of the questionnaire was then conducted with another small group of volunteers.” | We cannot locate where this sentence has been duplicated. The paragraph refers to the two stages of pre-testing – the first was conducted during the initial stage using a hard copy of the questionnaire and the second was conducted online using the Qualtrics platform. |
| **Section** | **Line(s)** | **Comment** | **Action taken** |
| Questionnaire design (continued) | 173-180 | What did you do with pre-tested data? Was it analysed? What was the reliability score of the Questionnaire? State the Cronbach's alpha score. | We have sought further advice from a University of Glasgow statistician, specialising into medical research, who has confirmed that Cronbach’s alpha has very limited use which does not apply to this study, since the survey questions were only completed once per participant. Relevant reference: “On the Use, the Misuse, and the Very Limited Usefulness of Cronbach’s Alpha” <https://pmc.ncbi.nlm.nih.gov/articles/PMC2792363/> |
| Data analysis | 195 | I understand the data were downloaded into Microsoft Excel. Did you also analyze the data with Excel? If No, state the software used for the analyses (especially the inferential statistics). | Statistical analysis was performed using the IBM SPSS software, version 29.0.1.0 (171). This detail has been added. |
| Results | 221-223 | State the p-value after this statement as it not advisable to draw conclusions on descriptive statistics, “... but VNS had increased probability than VS to have responsibility for multiple pet species (Fig 2).” | This statement has been reworded to reflect the fact that a greater number of VNS than VS had responsibility for multiple pet species, but the difference was not statistically significant. |
|  | 231-233 | State the p-value after this statement as it not advisable to draw conclusions on descriptive statistics “In general, VNS were more likely than VS to discuss their pets’ nutrition with a qualified veterinary nurse.” | This statement has been reworded to reflect that more VNS than VS had discussed their pets’ nutrition with a qualified veterinary nurse, but the difference was not statistically significant. |
