# Supplementary material for: A survey of UK and Irish veterinary and veterinary nursing Students’ engagement with small animal nutrition information
Source: PLoS One. 2026 Jul 16;21(7):e0351963. doi: 10.1371/journal.pone.0351963 (PMC13374976; doi:10.1371/journal.pone.0351963)
Supplement: Attachment — Submitted filename: Comments to AuthorsPONE_2.docx [file pone.0351963.s007.docx]

Methods

Study participants

Authors have stated the population size of 1,360 for veterinary nurse students (VNS), BUT failed to state the population size of veterinary students (VS).

Authors should state the sample size formula used for the calculation, whether the Cochrane formula, Slovin’s formula, etc. Otherwise, they should report the power of the study.

The margin of error of 6.5% for VS, and 5.4% for VNS, respectively, is notably high. Authors should discuss this in the limitations of the study.

Questionnaire design

The Authors seem to have misunderstood my comment on the reliability of the instrument used. I did not tell them to use the Cronbach's alpha score to test for consistency. They should use it to check the reliability of the Questionnaire.

In their response, authors referred to an article by Klaas Sijtsma on ‘On the Use, the Misuse, and the Very Limited Usefulness of Cronbach’s Alpha’. Authors should be reminded that Klaas Sijtsma stance on Cronbach's alpha score for reliability was not clear. They only said that Alpha is not a measure of internal consistency.

Klaas Sijtsma’s concluding remarks on Alpha as a measure of reliability is as follows, “Reporting alpha in addition to a greater lower bound may be a good strategy to introduce and promote a better reliability estimation practice.”
