# Supplementary material for: A survey of UK and Irish veterinary and veterinary nursing Students’ engagement with small animal nutrition information
Source: PLoS One. 2026 Jul 16;21(7):e0351963. doi: 10.1371/journal.pone.0351963 (PMC13374976; doi:10.1371/journal.pone.0351963)
Supplement: Attachment — Submitted filename: Response to Reviewers PONE-D-25-21707R3.docx [file pone.0351963.s009.docx]

# Response to reviewers in relation to manuscript ID: PONE-D-25-21707R3

# ‘A Survey of UK and Irish Veterinary and Veterinary Nursing Students’ Engagement with Small Animal Nutrition Information’.

The authors would like to thank the Academic Editor and the reviewers for the time and effort devoted to reviewing our manuscript. We appreciate the constructive comments, and we have revised the manuscript accordingly. Please find below details of how these comments have been addressed. The line numbers are correct according to the marked-up version of the revised manuscript.

| **Section** | **Line(s)** | **Comment** | **Action taken** |
| --- | --- | --- | --- |
| Methods  Study participants | 126-128 | Authors have stated the population size of 1,360 for veterinary nurse students (VNS), BUT failed to state the population size of veterinary students (VS). | The population size of veterinary students has been clarified. |
|  | 132-134 | Authors should state the sample size formula used for the calculation, whether the Cochrane formula, Slovin’s formula, etc. Otherwise, they should report the power of the study. | The sample size formula used for the calculation has been reported. |
| Questionnaire design | 180-181 and 379-380 | The authors should use Cronbach’s Alpha to check the reliability of the Questionnaire | The Cronbach’s Alpha score has been reported. |
| Limitations | 535-539 | The margin of error of 6.5% for VS, and 5.4% for VNS, respectively, is notably high. Authors should discuss this in the limitations of the study. | This has been discussed within the limitations of the study. |
