# Supplementary material for: A survey of UK and Irish veterinary and veterinary nursing Students’ engagement with small animal nutrition information
Source: PLoS One. 2026 Jul 16;21(7):e0351963. doi: 10.1371/journal.pone.0351963 (PMC13374976; doi:10.1371/journal.pone.0351963)
Supplement: Peer review history

# Peer review: Decision Letter 0

**DOI:** 10.1371/journal.pone.0351963.r001

**Authors:** Amr Zaineldin

8 Aug 2025

PONE-D-25-21707

Used without Trust: Student Veterinary Professionals’ Engagement with Small Animal Nutrition Information.

PLOS ONE

Dear Dr. Lumbis,

Thank you for submitting your manuscript to PLOS ONE. After careful consideration, we have decided that your manuscript does not meet our criteria for publication and must therefore be rejected.

Specifically:

The manuscript requires extensive revisions that fundamentally alter its structure, clarity, and focus. Given the depth of changes needed—particularly in methodology transparency, results presentation, and discussion framing.

I am sorry that we cannot be more positive on this occasion, but hope that you appreciate the reasons for this decision.

Kind regards,

Amr Zaineldin, Ph. D.

Academic Editor

PLOS ONE

Additional Editor Comments :

1-The title "Used without Trust" is misleading and should be revised to reflect the geographical scope and focus on information-seeking behavior.

2-Line 47: Clarify "key trend" with a reference.

3-Lines 50–51: Justify "veterinary breakthrough" claim.

4-Line 60: Rephrase "low desire" to reflect caregivers’ actual motivations.

5-Line 47: Add references.

6-Methedology: Lack of details on questionnaire design and specific questions analyzed.

7-Result: Misalignment between Methods and Results subsections,and overinterpretation of first-year students’ lack of confidence.

Reviewers' comments:

Reviewer's Responses to Questions

**Comments to the Author**

1. Is the manuscript technically sound, and do the data support the conclusions?

The manuscript must describe a technically sound piece of scientific research with data that supports the conclusions. Experiments must have been conducted rigorously, with appropriate controls, replication, and sample sizes. The conclusions must be drawn appropriately based on the data presented.

Reviewer #1: Partly

**********

2. Has the statistical analysis been performed appropriately and rigorously?

Reviewer #1: I Don't Know

**********

3. Have the authors made all data underlying the findings in their manuscript fully available?

The PLOS Data policy requires authors to make all data underlying the findings described in their manuscript fully available without restriction, with rare exception (please refer to the Data Availability Statement in the manuscript PDF file). The data should be provided as part of the manuscript or its supporting information, or deposited to a public repository. For example, in addition to summary statistics, the data points behind means, medians and variance measures should be available. If there are restrictions on publicly sharing data—e.g. participant privacy or use of data from a third party—those must be specified.

Reviewer #1: No

**********

4. Is the manuscript presented in an intelligible fashion and written in standard English?

PLOS ONE does not copyedit accepted manuscripts, so the language in submitted articles must be clear, correct, and unambiguous. Any typographical or grammatical errors should be corrected at revision, so please note any specific errors here.

Reviewer #1: Yes

**********

5. Review Comments to the Author

Please use the space provided to explain your answers to the questions above. You may also include additional comments for the author, including concerns about dual publication, research ethics, or publication ethics. (Please upload your review as an attachment if it exceeds 20,000 characters)

Reviewer #1: Title:

I found this misleading given that it is in reference to “small animal nutrition information”, implying all small animal nutrition information…that which might be trusted and untrusted. Suggest dropping the “used without trust” part of the title. Also, adding in the geographical locations of the study would be helpful, especially to compare with very similar studies conducted elsewhere (e.g. Canada and the United States).

Introduction:

Line 47: is there a reference to support the “key trend”? Also, the opening sentence is very long and cumbersome – suggest divide into two separate sentences to make the points clearer to follow.

Line 50 & 51: I don’t understand how “greater nutritional variety” is “a veterinary breakthrough for pets” – how so? And why? There are other things that are veterinary breakthroughs,

Line 60: I don’t think pet caregivers have a low desire to access reliable and credible information, in fact quite the opposite…I think they do desire such information, and reliable access is the issue.

Method:

Questionnaire design: how was the larger questionnaire designed? How did you decide on what questions to include in the questionnaire design?

The questions that were used for reporting purposes for this manuscript are vague. While there was mention made of the sections in the questionnaire (sections a-e), there was no detail provided on what the questions were. In fact the methodology section alluded to the idea that there was a larger questionnaire and a sub-set of responses were included in the present manuscript. Please provide detail on the actual questions that were asked and precisely which questions were included for this manuscript.

Results:

Line 190: it is not clear if the participants answered the questions in relation to one animal only or for all the animals that were under their care? Please elaborate how this was handled in the Methods section.

Line 243 & 244 and the paragraph that follows: it is not surprising that first year VS and VNS did not feel sufficient knowable to access evidenced based information themselves since they are likely at the very early stages of learning how to do this. The comments made here should be mitigated with the understanding that these students are an early stage of learning.

Related to the comments above for the Methodology section, it is unclear what results related to which questions. Can the authors provide the precise questions that were answered by the VS and VNS in the Methodology section and then subsequently present the data that was collected from these questions in the Results sections?

Using the same wording for both the questions in the Methods and sub-headings in the Results section will help the reader to match up questions with outcome data. Currently, there are two sub-headings used in the Results section that are vaguely related to the mentioned sections a-e in the questionnaire but not entirely clear as to how these sections relate to the questions that were presented to students.

Discussion:

Lines 407 – 410: I may have missed this aspect in the Results section, I did not see data that related to participants’ confidence in their ability to decipher information? The comments above for the Methods & Results section to make apparent what questions were asked and what their outcomes were, may help with this.

Overall comment for Discussion section: it seemed that most of the outcomes from this study were comparable to similar studies previously conducted for veterinary students and sources of nutrition information and perceptions of trust. The one unique aspect about this study is the inclusion of vet nursing students – I think the authors could expand the overall relevance and future impact of VNS perceptions and trust in information.

One important aspect that was missed in the Discussion is the fact that VS & VNS are also clients. They themselves own and care for animals making their perceptions and trust in nutrition information even more impactful for when they themselves are servicing clients as vet nurses and veterinarians. I would encourage the authors to consider discussing this aspect and relating the impact of this to the future roles of the participants.

**********

6. PLOS authors have the option to publish the peer review history of their article (what does this mean?). If published, this will include your full peer review and any attached files.

If you choose “no”, your identity will remain anonymous but your review may still be made public.

**Do you want your identity to be public for this peer review?**  For information about this choice, including consent withdrawal, please see our Privacy Policy.

Reviewer #1: No

**********

# Author comment: Author response to Decision Letter 1

**DOI:** 10.1371/journal.pone.0351963.r002

5 Sep 2025

We appreciate the time and effort invested by the reviewer and the editorial team in evaluating our research. Thank you for reinstating our submission and agreeing to conduct another editorial evaluation of our manuscript. We understand and acknowledge the concerns that have been raised and we have made the necessary revisions to address these and strengthen the manuscript accordingly. Please refer to the response to reviewers document which outlines a point-by-point response to the concerns raised and details of revisions carried out on the manuscript since its original submission.

Attachment Submitted filename: Response to reviewers PONE-D-25-21707.docx

# Peer review: Decision Letter 1

**DOI:** 10.1371/journal.pone.0351963.r003

**Authors:** Amr Zaineldin, Faham Khamesipour

19 Dec 2025

PONE-D-25-21707R1

A Survey of UK and Irish Veterinary and Veterinary Nursing Students’ Engagement with Small Animal Nutrition Information

PLOS One

Dear Dr. Lumbis,

Thank you for submitting your manuscript to PLOS ONE. After careful consideration, we feel that it has merit but does not fully meet PLOS ONE’s publication criteria as it currently stands. Therefore, we invite you to submit a revised version of the manuscript that addresses the points raised during the review process.

==============================

**ACADEMIC EDITOR:**

- Thank you for the revisions made to the manuscript so far. The reviewer’s suggestions are useful, and I recommend addressing the following points to further improve clarity and completeness:
  Abstract: In line 24, please use the possessive form of "study" to enhance grammatical accuracy.
  Introduction: For lines 102 to 105, the sentence “This manuscript presents results obtained as part of a broader longitudinal two-part project to evaluate and compare the small animal nutrition-related knowledge, attitudes and competence of foundation and professional phase veterinary students (VS) and veterinary nursing students (VNS)” could be paraphrased for clearer meaning. Please rephrase it for better readability.
  Methods: Lines 114 to 117 need rephrasing to clarify the sentence: “The study design and questionnaire was reviewed and given ethics approval by the University of Glasgow, College of Medical, Veterinary & Life Sciences Ethics Committee (reference number 200230001).” Kindly provide a clearer expression.
  Small Animals: The term “Small Animals” needs to be explicitly defined or explained within the context of this study. Please clarify what constitutes “Small Animals” for your readers.
  Study Participants: It is important to clearly describe how the sample size was determined. If no formula was used, please calculate and report the study's power.
  Questionnaire Design: Please include the internal consistency and reliability score of the questionnaire, such as the Cronbach’s alpha score, for transparency and methodological rigor.
  Data Collection: Specify what measures were put in place to ensure participants do not submit multiple responses.
  Data Analysis: Please mention the version of Microsoft Excel used for the data analysis in the Methods section for full transparency.
  Results (Pet Ownership): There are issues with the presentation of results. P-values like (p=<.015) and (p=<.021) should be professionally presented as (p < 0.015) or (p = 0.021). Kindly revise the statistical presentation for consistency and professionalism.
  Discussion: The Discussion section lacks a focus on the clinical implications of the results. Please include a section discussing how these findings could impact clinical practices or inform future research.
  Once these points are addressed, the manuscript will be significantly clearer and more comprehensive. Please make the necessary revisions and resubmit.

-->==============================

Please submit your revised manuscript by Feb 02 2026 11:59PM. If you will need more time than this to complete your revisions, please reply to this message or contact the journal office at plosone@plos.org. When you're ready to submit your revision, log on to https://www.editorialmanager.com/pone/ and select the 'Submissions Needing Revision' folder to locate your manuscript file.

Please include the following items when submitting your revised manuscript:

- A letter that responds to each point raised by the academic editor and reviewer(s). You should upload this letter as a separate file labeled 'Response to Reviewers'.
- A marked-up copy of your manuscript that highlights changes made to the original version. You should upload this as a separate file labeled 'Revised Manuscript with Track Changes'.
- An unmarked version of your revised paper without tracked changes. You should upload this as a separate file labeled 'Manuscript'.

-->If you would like to make changes to your financial disclosure, please include your updated statement in your cover letter. Guidelines for resubmitting your figure files are available below the reviewer comments at the end of this letter.

If applicable, we recommend that you deposit your laboratory protocols in protocols.io to enhance the reproducibility of your results. Protocols.io assigns your protocol its own identifier (DOI) so that it can be cited independently in the future. For instructions see: https://journals.plos.org/plosone/s/submission-guidelines#loc-laboratory-protocols. Additionally, PLOS ONE offers an option for publishing peer-reviewed Lab Protocol articles, which describe protocols hosted on protocols.io. Read more information on sharing protocols at https://plos.org/protocols?utm_medium=editorial-email&utm_source=authorletters&utm_campaign=protocols.

We look forward to receiving your revised manuscript.

Kind regards,

Faham Khamesipour, Ph.D.

Academic Editor

PLOS One

Journal Requirements:

When submitting your revision, we need you to address these additional requirements.

1. Please ensure that your manuscript meets PLOS ONE's style requirements, including those for file naming. The PLOS ONE style templates can be found at

https://journals.plos.org/plosone/s/file?id=wjVg/PLOSOne_formatting_sample_main_body.pdf and

https://journals.plos.org/plosone/s/file?id=ba62/PLOSOne_formatting_sample_title_authors_affiliations.pdf

2. Please provide additional details regarding participant consent. In the ethics statement in the Methods and online submission information, please ensure that you have specified (1) whether consent was informed and (2) what type you obtained (for instance, written or verbal, and if verbal, how it was documented and witnessed). If your study included minors, state whether you obtained consent from parents or guardians. If the need for consent was waived by the ethics committee, please include this information.

If you are reporting a retrospective study of medical records or archived samples, please ensure that you have discussed whether all data were fully anonymized before you accessed them and/or whether the IRB or ethics committee waived the requirement for informed consent. If patients provided informed written consent to have data from their medical records used in research, please include this information.

3. Please provide additional details regarding participant consent. In the ethics statement in the Methods and online submission information, please ensure that you have specified what type you obtained (for instance, written or verbal, and if verbal, how it was documented and witnessed). If your study included minors, state whether you obtained consent from parents or guardians. If the need for consent was waived by the ethics committee, please include this information.

4. Thank you for stating the following in the Acknowledgments Section of your manuscript:

“This paper has been produced as part of the first author’s doctoral degree which is being funded by the University of Glasgow, Small Animal Hospital Fund (The Ronald Miller Foundation).”

We note that you have provided funding information that is not currently declared in your Funding Statement. However, funding information should not appear in the Acknowledgments section or other areas of your manuscript. We will only publish funding information present in the Funding Statement section of the online submission form.

Please remove any funding-related text from the manuscript and let us know how you would like to update your Funding Statement. Currently, your Funding Statement reads as follows:

“This paper has been produced as part of the first author’s (RL) doctoral degree which is being funded by the University of Glasgow, Small Animal Hospital Fund (The Ronald Miller Foundation). The funders played no role in the study design, data collection and analysis, decision to publish, or preparation of the manuscript.”

Please include your amended statements within your cover letter; we will change the online submission form on your behalf.

5. We note that you have indicated that there are restrictions to data sharing for this study. For studies involving human research participant data or other sensitive data, we encourage authors to share de-identified or anonymized data. However, when data cannot be publicly shared for ethical reasons, we allow authors to make their data sets available upon request. For information on unacceptable data access restrictions, please see http://journals.plos.org/plosone/s/data-availability#loc-unacceptable-data-access-restrictions.

Before we proceed with your manuscript, please address the following prompts:

a) If there are ethical or legal restrictions on sharing a de-identified data set, please explain them in detail (e.g., data contain potentially identifying or sensitive patient information, data are owned by a third-party organization, etc.) and who has imposed them (e.g., a Research Ethics Committee or Institutional Review Board, etc.). Please also provide contact information for a data access committee, ethics committee, or other institutional body to which data requests may be sent.

b) If there are no restrictions, please upload the minimal anonymized data set necessary to replicate your study findings to a stable, public repository and provide us with the relevant URLs, DOIs, or accession numbers. Please see http://www.bmj.com/content/340/bmj.c181.long for guidelines on how to de-identify and prepare clinical data for publication. For a list of recommended repositories, please see https://journals.plos.org/plosone/s/recommended-repositories. You also have the option of uploading the data as Supporting Information files, but we would recommend depositing data directly to a data repository if possible.

Please update your Data Availability statement in the submission form accordingly.

6. Please include captions for your Supporting Information files at the end of your manuscript, and update any in-text citations to match accordingly. Please see our Supporting Information guidelines for more information: http://journals.plos.org/plosone/s/supporting-information.

If the reviewer comments include a recommendation to cite specific previously published works, please review and evaluate these publications to determine whether they are relevant and should be cited. There is no requirement to cite these works unless the editor has indicated otherwise.

Please review your reference list to ensure that it is complete and correct. If you have cited papers that have been retracted, please include the rationale for doing so in the manuscript text, or remove these references and replace them with relevant current references. Any changes to the reference list should be mentioned in the rebuttal letter that accompanies your revised manuscript. If you need to cite a retracted article, indicate the article’s retracted status in the References list and also include a citation and full reference for the retraction notice.

Additional Editor Comments (if provided):

[Note: HTML markup is below. Please do not edit.]

Reviewers' comments:

Reviewer's Responses to Questions

**Comments to the Author**

1. If the authors have adequately addressed your comments raised in a previous round of review and you feel that this manuscript is now acceptable for publication, you may indicate that here to bypass the “Comments to the Author” section, enter your conflict of interest statement in the “Confidential to Editor” section, and submit your "Accept" recommendation.

Reviewer #2: (No Response)

**********

2. Is the manuscript technically sound, and do the data support the conclusions?

The manuscript must describe a technically sound piece of scientific research with data that supports the conclusions. Experiments must have been conducted rigorously, with appropriate controls, replication, and sample sizes. The conclusions must be drawn appropriately based on the data presented.

Reviewer #2: Yes

**********

3. Has the statistical analysis been performed appropriately and rigorously?

Reviewer #2: Yes

**********

4. Have the authors made all data underlying the findings in their manuscript fully available?

The PLOS Data policy requires authors to make all data underlying the findings described in their manuscript fully available without restriction, with rare exception (please refer to the Data Availability Statement in the manuscript PDF file). The data should be provided as part of the manuscript or its supporting information, or deposited to a public repository. For example, in addition to summary statistics, the data points behind means, medians and variance measures should be available. If there are restrictions on publicly sharing data—e.g. participant privacy or use of data from a third party—those must be specified.

Reviewer #2: Yes

**********

5. Is the manuscript presented in an intelligible fashion and written in standard English?

PLOS ONE does not copyedit accepted manuscripts, so the language in submitted articles must be clear, correct, and unambiguous. Any typographical or grammatical errors should be corrected at revision, so please note any specific errors here.

Reviewer #2: No

**********

6. Review Comments to the Author

Please use the space provided to explain your answers to the questions above. You may also include additional comments for the author, including concerns about dual publication, research ethics, or publication ethics. (Please upload your review as an attachment if it exceeds 20,000 characters)

Reviewer #2: Abstract

Line 24 – Use the possessive form of “study”

Introduction

Lines 102 to 105: Paraphrase the following to make meaning clearer: “This manuscript presents results obtained as part of a broader longitudinal two-part project to evaluate and compare the small animal nutrition-related knowledge, attitudes and competence of foundation and professional phase veterinary students (VS) and veterinary nursing students (VNS).”

Methods

Lines 114 to 117: Paraphrase the following to make meaning clearer: “ The study design and questionnaire was reviewed and given ethics approval by the University of Glasgow, College of Medical, Veterinary & Life Sciences Ethics Committee (reference number 200230001).”

Authors must add what “Small Animals” mean or constitute in this study.

Study participants

Authors must clearly explained how they arrived at the sample size. If No formula was employed, they must calculate the power of the study.

Questionnaire design

What was the internal consistency and reliability score of the Questionnaire? State the Cronbach's alpha score.

Data collection

What mechanism was put in place to ensure that participants don’t submit two or more responses?

Data analysis

State the version of the Microsoft Excel used for the analyses.

Results

Pet ownership

Poor mathematical presentation of results. P-values such as (p=<.015), (p=<.021), etc must be presented professionally.

Discussion

The Discussion lacks clinical implication of results. Authors must include Clinical Implications.

**********

7. PLOS authors have the option to publish the peer review history of their article (what does this mean?). If published, this will include your full peer review and any attached files.

If you choose “no”, your identity will remain anonymous but your review may still be made public.

**Do you want your identity to be public for this peer review?**  For information about this choice, including consent withdrawal, please see our Privacy Policy.

Reviewer #2: No

**********

[NOTE: If reviewer comments were submitted as an attachment file, they will be attached to this email and accessible via the submission site. Please log into your account, locate the manuscript record, and check for the action link "View Attachments". If this link does not appear, there are no attachment files.]

To ensure your figures meet our technical requirements, please review our figure guidelines: https://journals.plos.org/plosone/s/figures

You may also use PLOS’s free figure tool, NAAS, to help you prepare publication quality figures: https://journals.plos.org/plosone/s/figures#loc-tools-for-figure-preparation.

NAAS will assess whether your figures meet our technical requirements by comparing each figure against our figure specifications.

Attachment Submitted filename: Comments to AuthorsPONE.docx

# Author comment: Author response to Decision Letter 2

**DOI:** 10.1371/journal.pone.0351963.r004

29 Jan 2026

We appreciate the comments provided by the Academic Editor and the reviewers. We have responded to these within the response to reviewers document.

Attachment Submitted filename: Response to Reviewers PONE-D-25-21707R1.docx

# Peer review: Decision Letter 2

**DOI:** 10.1371/journal.pone.0351963.r005

**Authors:** Amr Zaineldin, Faham Khamesipour, Faham Khamesipour

26 Feb 2026

PONE-D-25-21707R2

A Survey of UK and Irish Veterinary and Veterinary Nursing Students’ Engagement with Small Animal Nutrition Information

PLOS One

Dear Dr. Lumbis,

Thank you for submitting your manuscript to PLOS ONE. After careful consideration, we feel that it has merit but does not fully meet PLOS ONE’s publication criteria as it currently stands. Therefore, we invite you to submit a revised version of the manuscript that addresses the points raised during the review process.

==============================

**ACADEMIC EDITOR:**

- Following peer review, your manuscript has been considered suitable in scope and relevance; however, it requires minor revision before it can be accepted for publication. Please move the definition of “small animal” from the Introduction to the Methods section, provide the full form of RCVS at first mention, and clearly state the total population size of VS and VNS from which the sample was drawn. Scientifically justify the adequacy of the sample size, and if no formal calculation was performed, provide a post hoc power analysis. In the questionnaire design section, delete the repeated sentence regarding the pre-test, clarify how the pre-test data were handled, and report the reliability of the instrument, including the Cronbach’s alpha value. Additionally, specify the statistical software used for inferential analyses, as stating that data were downloaded into Excel is insufficient. In the Results section, ensure that all comparative statements particularly those comparing VS and VNS are supported with corresponding p-values, since conclusions should not be based solely on descriptive statistics. Please submit a revised manuscript along with a detailed response to all reviewer comments..
  ==============================
  Please submit your revised manuscript by Apr 12 2026 11:59PM. If you will need more time than this to complete your revisions, please reply to this message or contact the journal office at plosone@plos.org. When you're ready to submit your revision, log on to https://www.editorialmanager.com/pone/ and select the 'Submissions Needing Revision' folder to locate your manuscript file.
  Please include the following items when submitting your revised manuscript:

- A letter that responds to each point raised by the academic editor and reviewer(s). You should upload this letter as a separate file labeled 'Response to Reviewers'.
- A marked-up copy of your manuscript that highlights changes made to the original version. You should upload this as a separate file labeled 'Revised Manuscript with Track Changes'.
- An unmarked version of your revised paper without tracked changes. You should upload this as a separate file labeled 'Manuscript'.

-->If you would like to make changes to your financial disclosure, please include your updated statement in your cover letter. Guidelines for resubmitting your figure files are available below the reviewer comments at the end of this letter.

If applicable, we recommend that you deposit your laboratory protocols in protocols.io to enhance the reproducibility of your results. Protocols.io assigns your protocol its own identifier (DOI) so that it can be cited independently in the future. For instructions see: https://journals.plos.org/plosone/s/submission-guidelines#loc-laboratory-protocols. Additionally, PLOS ONE offers an option for publishing peer-reviewed Lab Protocol articles, which describe protocols hosted on protocols.io. Read more information on sharing protocols at https://plos.org/protocols?utm_medium=editorial-email&utm_source=authorletters&utm_campaign=protocols.

We look forward to receiving your revised manuscript.

Kind regards,

Faham Khamesipour, Ph.D.

Academic Editor

PLOS One

Journal Requirements:

If the reviewer comments include a recommendation to cite specific previously published works, please review and evaluate these publications to determine whether they are relevant and should be cited. There is no requirement to cite these works unless the editor has indicated otherwise.

Please review your reference list to ensure that it is complete and correct. If you have cited papers that have been retracted, please include the rationale for doing so in the manuscript text, or remove these references and replace them with relevant current references. Any changes to the reference list should be mentioned in the rebuttal letter that accompanies your revised manuscript. If you need to cite a retracted article, indicate the article’s retracted status in the References list and also include a citation and full reference for the retraction notice.

[Note: HTML markup is below. Please do not edit.]

Reviewers' comments:

Reviewer's Responses to Questions

**Comments to the Author**

1. If the authors have adequately addressed your comments raised in a previous round of review and you feel that this manuscript is now acceptable for publication, you may indicate that here to bypass the “Comments to the Author” section, enter your conflict of interest statement in the “Confidential to Editor” section, and submit your "Accept" recommendation.

Reviewer #2: (No Response)

**********

2. Is the manuscript technically sound, and do the data support the conclusions?

The manuscript must describe a technically sound piece of scientific research with data that supports the conclusions. Experiments must have been conducted rigorously, with appropriate controls, replication, and sample sizes. The conclusions must be drawn appropriately based on the data presented.

Reviewer #2: Partly

**********

3. Has the statistical analysis been performed appropriately and rigorously?

Reviewer #2: Yes

**********

4. Have the authors made all data underlying the findings in their manuscript fully available?

The PLOS Data policy requires authors to make all data underlying the findings described in their manuscript fully available without restriction, with rare exception (please refer to the Data Availability Statement in the manuscript PDF file). The data should be provided as part of the manuscript or its supporting information, or deposited to a public repository. For example, in addition to summary statistics, the data points behind means, medians and variance measures should be available. If there are restrictions on publicly sharing data—e.g. participant privacy or use of data from a third party—those must be specified.

Reviewer #2: Yes

**********

5. Is the manuscript presented in an intelligible fashion and written in standard English?

PLOS ONE does not copyedit accepted manuscripts, so the language in submitted articles must be clear, correct, and unambiguous. Any typographical or grammatical errors should be corrected at revision, so please note any specific errors here.

Reviewer #2: Yes

**********

6. Review Comments to the Author

Please use the space provided to explain your answers to the questions above. You may also include additional comments for the author, including concerns about dual publication, research ethics, or publication ethics. (Please upload your review as an attachment if it exceeds 20,000 characters)

Reviewer #2: Introduction

Lines 105-107: Move the following statement to the Methods section “For the purposes of this study, small animal was defined as companion animals or pets, kept for domestic purposes rather than for food production.”

Methods

Study participants

Line 122; Write full form of “RCVS”

Information on how the sample size was obtained is scanty. We need to know whether the sample size meets the minimum requirement for scientific inferences. First, give us a general idea about the total number of VS and VNS in the selected schools. Please state the population size of both VS and VNS from which you recruited the 135 veterinary students (VS) and 186 veterinary nursing students. Scientifically justify why the sample size is good to make general conclusions about the population. If no formula was employed, the authors must calculate the power of the study.

Questionnaire design

Lines 162-163: This sentence has already been stated above. Delete it. “An online pre-test of the questionnaire was then conducted with another small group of volunteers.”

What did you do with pre-tested data? Was it analysed? What was the reliability score of the Questionnaire? State the Cronbach's alpha score.

Data Analysis

I understand the data were downloaded into Microsoft Excel. Did you also analyze the data with Excel? If No, state the software used for the analyses (especially the inferential statistics).

Results

Pet ownership

Lines 203-205; State the p-value after this statement as it not advisable to draw conclusions on descriptive statistics, “... but VNS had increased probability than VS to have responsibility for multiple pet species (Fig 2).”

Lines 212-213: State the p-value after this statement as it not advisable to draw conclusions on descriptive statistics “ In general, VNS were more likely than VS to discuss their pets’ nutrition with a qualified veterinary nurse.”

**********

7. PLOS authors have the option to publish the peer review history of their article (what does this mean?). If published, this will include your full peer review and any attached files.

If you choose “no”, your identity will remain anonymous but your review may still be made public.

**Do you want your identity to be public for this peer review?**  For information about this choice, including consent withdrawal, please see our Privacy Policy.

Reviewer #2: No

**********

[NOTE: If reviewer comments were submitted as an attachment file, they will be attached to this email and accessible via the submission site. Please log into your account, locate the manuscript record, and check for the action link "View Attachments". If this link does not appear, there are no attachment files.]

To ensure your figures meet our technical requirements, please review our figure guidelines: https://journals.plos.org/plosone/s/figures

You may also use PLOS’s free figure tool, NAAS, to help you prepare publication quality figures: https://journals.plos.org/plosone/s/figures#loc-tools-for-figure-preparation.

NAAS will assess whether your figures meet our technical requirements by comparing each figure against our figure specifications.

# Author comment: Author response to Decision Letter 3

**DOI:** 10.1371/journal.pone.0351963.r006

2 Apr 2026

Thank you for your time and comments, and for the opportunity to strengthen our publication. Please find our revised manuscript and responses to the reviewers’ comments for your consideration.

Attachment Submitted filename: Response to Reviewers PONE-D-25-21707R2.docx

# Peer review: Decision Letter 3

**DOI:** 10.1371/journal.pone.0351963.r007

**Authors:** Amr Zaineldin, Faham Khamesipour, Faham Khamesipour, Faham Khamesipour

1 May 2026

PONE-D-25-21707R3

A Survey of UK and Irish Veterinary and Veterinary Nursing Students’ Engagement with Small Animal Nutrition Information

PLOS One

Dear Dr. Lumbis,

Thank you for submitting your manuscript to PLOS ONE. After careful consideration, we feel that it has merit but does not fully meet PLOS ONE’s publication criteria as it currently stands. Therefore, we invite you to submit a revised version of the manuscript that addresses the points raised during the review process.

==============================

**ACADEMIC EDITOR:**

Thank you for the revisions made to the manuscript so far. The reviewer’s suggestions are useful, and I recommend addressing the following points to further improve clarity and completeness:

Methods

Study participants

Authors have stated the population size of 1,360 for veterinary nurse students (VNS), BUT failed to state the population size of veterinary students (VS).

Authors should state the sample size formula used for the calculation, whether the Cochrane formula, Slovin’s formula, etc. Otherwise, they should report the power of the study.

The margin of error of 6.5% for VS, and 5.4% for VNS, respectively, is notably high. Authors should discuss this in the limitations of the study.

Questionnaire design

The Authors seem to have misunderstood my comment on the reliability of the instrument used. I did not tell them to use the Cronbach's alpha score to test for consistency. They should use it to check the reliability of the Questionnaire.

In their response, authors referred to an article by Klaas Sijtsma on ‘On the Use, the Misuse, and the Very Limited Usefulness of Cronbach’s Alpha’. Authors should be reminded that Klaas Sijtsma stance on Cronbach's alpha score for reliability was not clear. They only said that Alpha is not a measure of internal consistency.

Klaas Sijtsma’s concluding remarks on Alpha as a measure of reliability is as follows, “Reporting alpha in addition to a greater lower bound may be a good strategy to introduce and promote a better reliability estimation practice.

==============================

Please submit your revised manuscript by Jun 15 2026 11:59PM. If you will need more time than this to complete your revisions, please reply to this message or contact the journal office at plosone@plos.org. When you're ready to submit your revision, log on to https://www.editorialmanager.com/pone/ and select the 'Submissions Needing Revision' folder to locate your manuscript file.

Please include the following items when submitting your revised manuscript:

- A letter that responds to each point raised by the academic editor and reviewer(s). You should upload this letter as a separate file labeled 'Response to Reviewers'.
- A marked-up copy of your manuscript that highlights changes made to the original version. You should upload this as a separate file labeled 'Revised Manuscript with Track Changes'.
- An unmarked version of your revised paper without tracked changes. You should upload this as a separate file labeled 'Manuscript'.

If you would like to make changes to your financial disclosure, please include your updated statement in your cover letter. Guidelines for resubmitting your figure files are available below the reviewer comments at the end of this letter.

If applicable, we recommend that you deposit your laboratory protocols in protocols.io to enhance the reproducibility of your results. Protocols.io assigns your protocol its own identifier (DOI) so that it can be cited independently in the future. For instructions see: https://journals.plos.org/plosone/s/submission-guidelines#loc-laboratory-protocols. Additionally, PLOS ONE offers an option for publishing peer-reviewed Lab Protocol articles, which describe protocols hosted on protocols.io. Read more information on sharing protocols at https://plos.org/protocols?utm_medium=editorial-email&utm_source=authorletters&utm_campaign=protocols.

As the corresponding author, your ORCID iD is verified in the submission system and will appear in the published article. PLOS supports the use of ORCID, and we encourage all coauthors to register for an ORCID iD and use it as well. Please encourage your coauthors to verify their ORCID iD within the submission system before final acceptance, as unverified ORCID iDs will not appear in the published article. *Only*  the individual author can complete the verification step; PLOS staff *cannot*  verify ORCID iDs on behalf of authors.

We look forward to receiving your revised manuscript.

Kind regards,

Faham Khamesipour, Ph.D.

Academic Editor

PLOS One

**Journal Requirements:**

1. If the reviewer comments include a recommendation to cite specific previously published works, please review and evaluate these publications to determine whether they are relevant and should be cited. There is no requirement to cite these works unless the editor has indicated otherwise.

2. Please review your reference list to ensure that it is complete and correct. If you have cited papers that have been retracted, please include the rationale for doing so in the manuscript text, or remove these references and replace them with relevant current references. Any changes to the reference list should be mentioned in the rebuttal letter that accompanies your revised manuscript. If you need to cite a retracted article, indicate the article’s retracted status in the References list and also include a citation and full reference for the retraction notice.

[Note: HTML markup is below. Please do not edit.]

Reviewers' comments:

Reviewer's Responses to Questions

**Comments to the Author**

1. If the authors have adequately addressed your comments raised in a previous round of review and you feel that this manuscript is now acceptable for publication, you may indicate that here to bypass the “Comments to the Author” section, enter your conflict of interest statement in the “Confidential to Editor” section, and submit your "Accept" recommendation.

Reviewer #2: (No Response)

**********

2. Is the manuscript technically sound, and do the data support the conclusions?

The manuscript must describe a technically sound piece of scientific research with data that supports the conclusions. Experiments must have been conducted rigorously, with appropriate controls, replication, and sample sizes. The conclusions must be drawn appropriately based on the data presented.

Reviewer #2: (No Response)

**********

3. Has the statistical analysis been performed appropriately and rigorously?

Reviewer #2: No

**********

4. Have the authors made all data underlying the findings in their manuscript fully available?

The PLOS Data policy requires authors to make all data underlying the findings described in their manuscript fully available without restriction, with rare exception (please refer to the Data Availability Statement in the manuscript PDF file). The data should be provided as part of the manuscript or its supporting information, or deposited to a public repository. For example, in addition to summary statistics, the data points behind means, medians and variance measures should be available. If there are restrictions on publicly sharing data—e.g. participant privacy or use of data from a third party—those must be specified.

Reviewer #2: No

**********

5. Is the manuscript presented in an intelligible fashion and written in standard English?

PLOS ONE does not copyedit accepted manuscripts, so the language in submitted articles must be clear, correct, and unambiguous. Any typographical or grammatical errors should be corrected at revision, so please note any specific errors here.

Reviewer #2: Yes

**********

6. Review Comments to the Author

Please use the space provided to explain your answers to the questions above. You may also include additional comments for the author, including concerns about dual publication, research ethics, or publication ethics. (Please upload your review as an attachment if it exceeds 20,000 characters)

Reviewer #2: Methods

Study participants

Authors have stated the population size of 1,360 for veterinary nurse students (VNS), BUT failed to state the population size of veterinary students (VS).

Authors should state the sample size formula used for the calculation, whether the Cochrane formula, Slovin’s formula, etc. Otherwise, they should report the power of the study.

The margin of error of 6.5% for VS, and 5.4% for VNS, respectively, is notably high. Authors should discuss this in the limitations of the study.

Questionnaire design

The Authors seem to have misunderstood my comment on the reliability of the instrument used. I did not tell them to use the Cronbach's alpha score to test for consistency. They should use it to check the reliability of the Questionnaire.

In their response, authors referred to an article by Klaas Sijtsma on ‘On the Use, the Misuse, and the Very Limited Usefulness of Cronbach’s Alpha’. Authors should be reminded that Klaas Sijtsma stance on Cronbach's alpha score for reliability was not clear. They only said that Alpha is not a measure of internal consistency.

Klaas Sijtsma’s concluding remarks on Alpha as a measure of reliability is as follows, “Reporting alpha in addition to a greater lower bound may be a good strategy to introduce and promote a better reliability estimation practice.”

**********

7. PLOS authors have the option to publish the peer review history of their article (what does this mean?). If published, this will include your full peer review and any attached files.

If you choose “no”, your identity will remain anonymous but your review may still be made public.

**Do you want your identity to be public for this peer review?**  For information about this choice, including consent withdrawal, please see our Privacy Policy.

Reviewer #2: No

**********

[NOTE: If reviewer comments were submitted as an attachment file, they will be attached to this email and accessible via the submission site. Please log into your account, locate the manuscript record, and check for the action link "View Attachments". If this link does not appear, there are no attachment files.]

To ensure your figures meet our technical requirements, please review our figure guidelines: https://journals.plos.org/plosone/s/figures

You may also use PLOS’s free figure tool, NAAS, to help you prepare publication quality figures: https://journals.plos.org/plosone/s/figures#loc-tools-for-figure-preparation.

NAAS will assess whether your figures meet our technical requirements by comparing each figure against our figure specifications.

Attachment Submitted filename: Comments to AuthorsPONE_2.docx

# Author comment: Author response to Decision Letter 4

**DOI:** 10.1371/journal.pone.0351963.r008

20 May 2026

Please refer to the response to reviewers document for details of how each comment has been addressed within the revised manuscript.

Attachment Submitted filename: Response to Reviewers PONE-D-25-21707R3.docx

# Peer review: Decision Letter 4

**DOI:** 10.1371/journal.pone.0351963.r009

**Authors:** Amr Zaineldin, Faham Khamesipour, Faham Khamesipour, Faham Khamesipour, Faham Khamesipour

4 Jun 2026

A Survey of UK and Irish Veterinary and Veterinary Nursing Students’ Engagement with Small Animal Nutrition Information

PONE-D-25-21707R4

Dear Dr. Lumbis,

We’re pleased to inform you that your manuscript has been judged scientifically suitable for publication and will be formally accepted for publication once it meets all outstanding technical requirements.

Within one week, you’ll receive an e-mail detailing the required amendments. When these have been addressed, you’ll receive a formal acceptance letter and your manuscript will be scheduled for publication.

An invoice will be generated when your article is formally accepted. Please note, if your institution has a publishing partnership with PLOS and your article meets the relevant criteria, all or part of your publication costs will be covered. Please make sure your user information is up-to-date by logging into Editorial Manager at Editorial Manager® and clicking the ‘Update My Information' link at the top of the page. For questions related to billing, please contact billing support.

If your institution or institutions have a press office, please notify them about your upcoming paper to help maximize its impact. If they’ll be preparing press materials, please inform our press team as soon as possible -- no later than 48 hours after receiving the formal acceptance. Your manuscript will remain under strict press embargo until 2 pm Eastern Time on the date of publication. For more information, please contact onepress@plos.org.

Kind regards,

Faham Khamesipour, Ph.D.

Academic Editor

PLOS One

Additional Editor Comments (optional):

Reviewers' comments:

Reviewer's Responses to Questions

**Comments to the Author**

1. If the authors have adequately addressed your comments raised in a previous round of review and you feel that this manuscript is now acceptable for publication, you may indicate that here to bypass the “Comments to the Author” section, enter your conflict of interest statement in the “Confidential to Editor” section, and submit your "Accept" recommendation.

Reviewer #2: All comments have been addressed

**********

2. Is the manuscript technically sound, and do the data support the conclusions?

The manuscript must describe a technically sound piece of scientific research with data that supports the conclusions. Experiments must have been conducted rigorously, with appropriate controls, replication, and sample sizes. The conclusions must be drawn appropriately based on the data presented.

Reviewer #2: Yes

**********

3. Has the statistical analysis been performed appropriately and rigorously?

Reviewer #2: Yes

**********

4. Have the authors made all data underlying the findings in their manuscript fully available?

The PLOS Data policy requires authors to make all data underlying the findings described in their manuscript fully available without restriction, with rare exception (please refer to the Data Availability Statement in the manuscript PDF file). The data should be provided as part of the manuscript or its supporting information, or deposited to a public repository. For example, in addition to summary statistics, the data points behind means, medians and variance measures should be available. If there are restrictions on publicly sharing data—e.g. participant privacy or use of data from a third party—those must be specified.

Reviewer #2: Yes

**********

5. Is the manuscript presented in an intelligible fashion and written in standard English?

PLOS ONE does not copyedit accepted manuscripts, so the language in submitted articles must be clear, correct, and unambiguous. Any typographical or grammatical errors should be corrected at revision, so please note any specific errors here.

Reviewer #2: No

**********

6. Review Comments to the Author

Please use the space provided to explain your answers to the questions above. You may also include additional comments for the author, including concerns about dual publication, research ethics, or publication ethics. (Please upload your review as an attachment if it exceeds 20,000 characters)

Reviewer #2: The authors have satisfactorily addressed the comments raised during the review process, and the manuscript has been substantially improved. However, the manuscript would benefit from professional English language editing to enhance clarity, grammar, readability, and overall presentation prior to publication.

**********

7. PLOS authors have the option to publish the peer review history of their article (what does this mean?). If published, this will include your full peer review and any attached files.

If you choose “no”, your identity will remain anonymous but your review may still be made public.

**Do you want your identity to be public for this peer review?**  For information about this choice, including consent withdrawal, please see our Privacy Policy.

Reviewer #2: No

**********

# Editor report: Acceptance letter

**DOI:** 10.1371/journal.pone.0351963.r010

**Authors:** Amr Zaineldin, Faham Khamesipour, Faham Khamesipour, Faham Khamesipour, Faham Khamesipour

PONE-D-25-21707R4

PLOS One

Dear Dr. Lumbis,

I'm pleased to inform you that your manuscript has been deemed suitable for publication in PLOS One. Congratulations! Your manuscript is now being handed over to our production team.

At this stage, our production department will prepare your paper for publication. This includes ensuring the following:

* All references, tables, and figures are properly cited

* All relevant supporting information is included in the manuscript submission,

* There are no issues that prevent the paper from being properly typeset

You will receive further instructions from the production team, including instructions on how to review your proof when it is ready. Please keep in mind that we are working through a large volume of accepted articles, so please give us a few days to review your paper and let you know the next and final steps.

Lastly, if your institution or institutions have a press office, please let them know about your upcoming paper now to help maximize its impact. If they'll be preparing press materials, please inform our press team within the next 48 hours. Your manuscript will remain under strict press embargo until 2 pm Eastern Time on the date of publication. For more information, please contact onepress@plos.org.

You will receive an invoice from PLOS for your publication fee after your manuscript has reached the completed accept phase. If you receive an email requesting payment before acceptance or for any other service, this may be a phishing scheme. Learn how to identify phishing emails and protect your accounts at https://explore.plos.org/phishing.

If we can help with anything else, please email us at customercare@plos.org.

Thank you for submitting your work to PLOS ONE and supporting open access.

Kind regards,

PLOS ONE Editorial Office Staff

on behalf of

Dr. Faham Khamesipour

Academic Editor

PLOS One
